# CO_2_ Hydrogenation Using Nonthermal Plasma: Effects of Packed Beds on Plasma Discharge Behavior

**DOI:** 10.1021/acs.iecr.5c04396

**Published:** 2026-02-23

**Authors:** Sathya M. Perera, Berkay Ekinci, Sven G. Bilén, Sean D. Knecht, Gina Noh

**Affiliations:** a Department of Chemistry, 8082 The Pennsylvania State University, University Park, Pennsylvania 16802, United States; b School of Electrical Engineering and Computer Science, 8082 The Pennsylvania State University, University Park, Pennsylvania 16802, United States; c School of Engineering Design and Innovation, 8082 The Pennsylvania State University, University Park, Pennsylvania 16802, United States; d Department of Chemical Engineering, 8082 The Pennsylvania State University, University Park, Pennsylvania 16802, United States

## Abstract

Nonthermal plasma-assisted catalysis offers a promising approach to activate strong chemical bonds such as those in CO_2_ and transform such stable molecules into value-added products under ambient conditions. Advancement in this field requires building upon understanding of plasma–catalyst interactions that dictate product selectivity, conversion, and energy efficiency. This study aims to systematically assess how different dielectric supports influence plasma discharge behavior and reactor performance during CO_2_ hydrogenation in a dielectric barrier discharge reactor. The dielectric constant of the packing material strongly influences discharge behavior and, consequently, catalytic activity. Among the materials examined, Al_2_O_3_ exhibited the highest CO_2_ conversion of 42% at a plasma power of 23 W, whereas CeO_2_ yielded the lowest (∼5%) due to the formation of a diffuse discharge, in contrast to localized discharges or surface streamers. CO_2_ conversion over CeO_2_ beds of a given particle size were nearly identical irrespective of changes in particle size, further supporting this discharge-dependent behavior. Notably, this work highlights the importance of comparing reactor performance for packed beds under nonpartial discharge operation.

## Graphical abstract

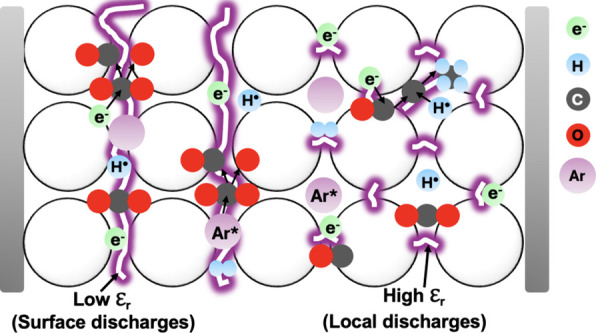

## 1 Introduction

CO_2_ valorization to fuels and platform chemicals^1–8^ is a promising strategy to mitigate anthropogenic CO_2_ emissions and close the carbon cycle.^9^ CO_2_ activation, even in the presence of heterogeneous catalysts, is thermodynamically and kinetically challenging,^10^ requiring operation of thermal catalytic processes at high temperatures and/or pressures.^11^ Nonthermal plasma (NTP) offers a promising alternative for activating strong chemical bonds, such as those in CO_2_.^12–14^ Additionally, NTP systems are compatible with intermittent excess renewable energy.^15^

NTP is a partially ionized gas in which energetic electrons generated by electric discharges collide with heavier gas atoms and molecules to produce reactive species including ions, radicals, and excited molecules. These species enable alternate reaction pathways that are often inaccessible under conventional thermal catalytic conditions. The key feature of NTP is its nonequilibrium nature, where electron temperatures (typically 10^4^–10^5^ K) are significantly higher than that of the bulk gas (200–1000 K).^16^ Several types of plasma reactors have been investigated for CO_2_ utilization.^17–22^ Of these, the dielectric barrier discharge (DBD) reactors are attractive because of their straightforward design, their compatibility with heterogeneous catalysts, and their operation at atmospheric pressure and in the absence of external heating.^23–29^

Research on plasma-enabled CO_2_ conversion includes CO_2_ dissociation to CO and O_2_, dry reforming of CH_4_ with CO_2_ to produce syngas (CO and H_2_) and liquid oxygenates, and CO_2_ hydrogenation to CO, CH_4_, C_2+_, and liquid oxygenates.^30–35^ Some studies are conducted with the presence of an inert gas, such as Ar, that decreases the plasma breakdown voltage and enhances CO_2_ activation through energy transfer and increased electron density.^36,37^ NTP coupled with heterogeneous catalysts located in the discharge region has resulted in improvements in reactor performance (reactant conversion, product selectivities, and electrical energy efficiency) compared to NTP alone.^38,39^ Zeng et al. reported that coupling NTP with Ni-K/Al_2_O_3_ at 160 °C yielded the best performance, significantly increasing the conversions of CO_2_ and CH_4_ and the yields of H_2_, CO, and C_2_–C_4_ paraffins and improving the energy efficiency of the process relative to plasma-only and catalyst-only systems.^40^ Similarly, Wang et al. found that CO_2_ hydrogenation in an empty DBD reactor predominantly produced CO (selectivity >80%). When a ^15^Co/Al_2_O_3_ was introduced, the methane selectivity increased from 3 to 45%, while CO selectivity decreased to 38% under ambient conditions. Furthermore, an optimized catalyst-bed configuration with 2 cm of ^15^Co/Al_2_O_3_ layered atop 3 cm of Al_2_O_3_ achieved a high C_2+_ hydrocarbon selectivity of 46% at CO_2_ conversion exceeding 70%.^41^ However, mechanistic understanding of plasma–catalyst interactions remains limited due to the intricate coupling of gas-phase and surface reactions, including electron-driven processes, adsorption–desorption dynamics, and discharge behavior.^42–44^ Solid materials, such as metal nanoparticles, dispersed on high surface area metal oxides that are typical examples of heterogeneous catalysts, introduced into the discharge region, alter the electric field distribution and plasma discharge behavior, which in turn impacts reactor performance.^45–48^

Metal oxides themselves, even in the absence of the active sites that render them heterogeneous catalysts, are reported to influence plasma discharge behavior. The dielectric constant and size of the packing material play key roles in determining plasma behavior by influencing how discharges form and propagate within the voids of the packed bed.^49–51^ Van Laer and Bogaerts reported that, at low dielectric constants, the plasma tends to form full-gap discharges with high electron density but low electric field strength and electron temperature while increasing the dielectric constant concentrates the electric field near particle contact points, producing localized discharges with higher field strength and electron temperature but reduced electron density due to enhanced wall losses.^52^ Similarly, Butterworth et al. also demonstrated that the dielectric constant of packing material strongly influences plasma behavior in a single-pellet DBD reactor.^53^ CO_2_ dissociation over packed beds of varied oxides has been studied in the presence of Ar^36^ and in its absence.^54^

Our work builds upon previous studies examining the effects of dielectric materials on discharge characteristics for plasma-enhanced CO_2_ dissociation^55,56^ and CH_4_ dry reforming.^57^ In this work, we examine how dielectric support materials influence discharge behavior and, in turn, CO_2_ hydrogenation performance. We examine CO_2_ hydrogenation to CO and CH_4_ in a coaxial DBD reactor, with and without oxides of varied dielectric constant at the same particle size, and for beds of CeO_2_ of varied particle sizes. CeO_2_ was selected for the particle size study to add to previous literature that examines the effects of particle sizes of Al_2_O_3_ and BaTiO_3_.^36^ CeO_2_ is particularly of interest because it is a catalyst support with distinct redox and oxygen-vacancy chemistries.^58–60^ Interestingly, under the operating conditions of our study, the performance of CeO_2_ does not depend on particle size and demonstrates the lowest activity in this study compared to Al_2_O_3_, TiO_2_, and BaTiO_3_. This performance is associated with the formation of a weak, homogeneous plasma, highlighting the importance of understanding reactor performance and intrinsic discharge behavior of support alone for any differences in configuration of the dielectric barrier, and the gas compositions prior to assessing the catalytic activity in plasma-catalytic systems.

## 2 Methods and Materials

### 2.1 Preparation and Characterization of Oxides

Three commercial-grade oxides were used as received without any prior treatment: γ-Al_2_O_3_ (Sasol PURALOX TH 100/150), TiO_2_ (Evonik AEROXIDE P90), and BaTiO_3_ (STANFORD Advanced Materials, 99.9%). CeO_2_ was prepared by calcining cerium­(III) nitrate hexahydrate (Sigma-Aldrich, 99%) in stagnant ambient air at 500 °C (2 °C/min, 2 h at 500 °C) in a muffle furnace (Thermolyne 48000). The specific surface area (from BET analysis), pore volume (single-point adsorption at *P*/*P* ^0^ ≈ 0.99), and average pore size (BJH desorption average pore width) of all oxide samples were determined by N_2_ physisorption at 77 K using a TriStar II Plus (Micrometrics) surface area and porosity analyzer, after degassing at 423 K overnight. All oxides were pressed (Carver Laboratory Press, 10,000 psi) and sieved to retain aggregates of these respective sizes: 180–250, 350–500, 600–1000, and 1400–1700 μm. The mass required to pack 5 cm of the fixed annular region of the DBD varied with material: 1.5 g for Al_2_O_3_, 4 g for CeO_2_, 2.8 g for TiO_2_, and 6.25 g for BaTiO_3_. The reported dielectric constants for γ-Al_2_O_3_, CeO_2_, and TiO_2_ are 9, 25, and 80, respectively.^60,61^ The measured dielectric constant for BaTiO_3_ is 6200–7000 (at Curie Point), as reported by the supplier.

### 2.2 Dielectric Barrier Discharge Reactor for Plasma-Enhanced CO_2_ Hydrogenation Reactions

CO_2_ conversion and selectivities for CO and hydrocarbons were measured for an annular dielectric barrier discharge (DBD) reactor with plug-flow hydrodynamics (schematically depicted in Figure 1). The high-voltage tungsten electrode (3 mm diameter) is axially aligned at the center of two concentric quartz tubes (1 mm wall thickness; 4 mm inner diameter for the inner tube and 10 mm inner diameter for the outer tube), around the outer of which is placed the ground electrode (aluminum foil; 50 mm in length). The region between the concentric quartz tubes (2.5 cm^3^) is the annulus (2 mm discharge gap; 50 mm length axial discharge region) through which gas mixtures flow and packed beds may be affixed, supported on packed glass wool.

**Figure 1:**
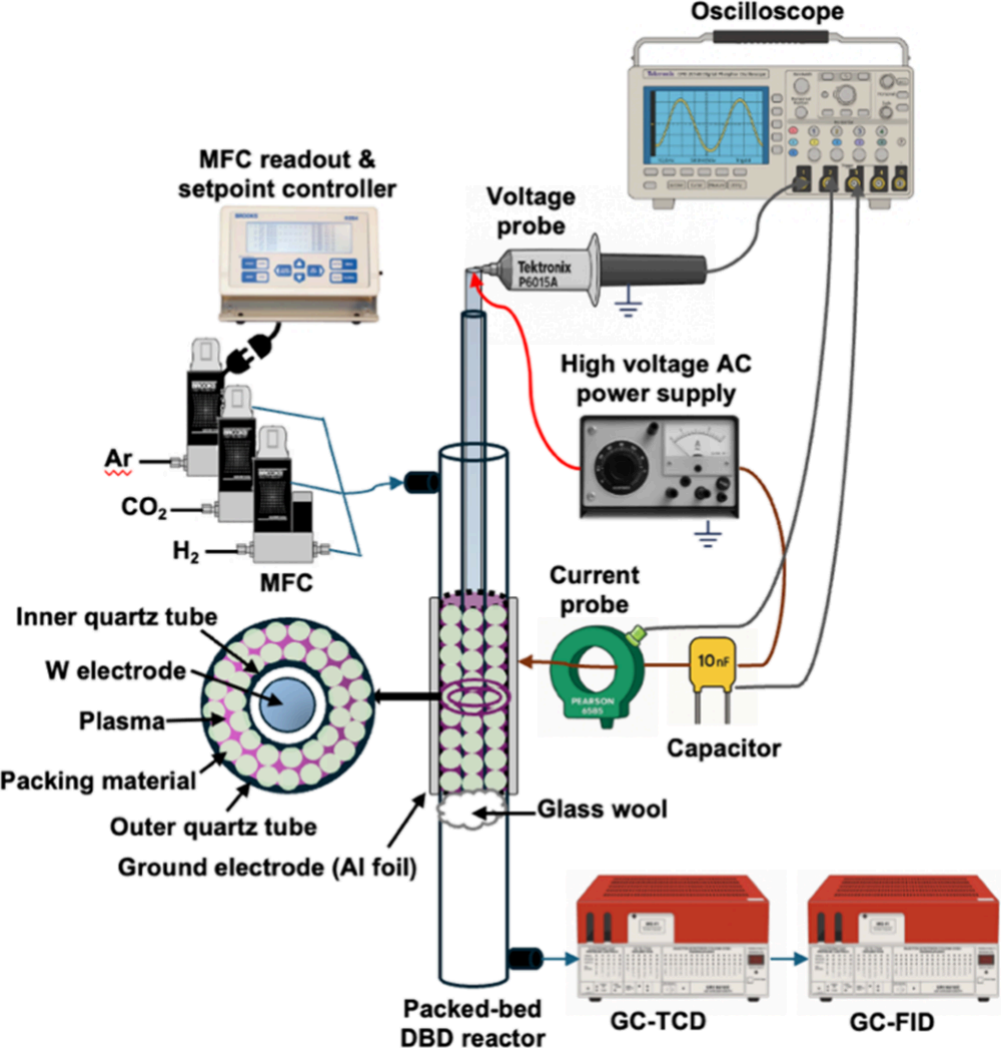
Schematic of the experimental setup. Gas flow paths are indicated by blue arrows, high-voltage electrical connection to the reactor is shown in red arrow, and ground connection is shown in brown arrow, and gray arrows denote the measurements of electrical signals.

A sinusoidal AC voltage of 23.5 kHz with a peak-to-peak amplitude of 12–24 kV was applied using a high-voltage power supply (PVM500 PLASMA DRIVER). Plasma power was determined via the Lissajous method by integrating the voltage signal recorded with a digital oscilloscope (Tektronix DPO 2014B, 100 MHz, 1 GS/s). High-voltage measurements were obtained with a Tektronix P6015A probe, while a reference capacitor (10 nF) connected in series for Lissajous measurements and a current probe (Pearson 6585) measures the instantaneous current and plasma voltage (discussed in Section 2.3). A 10X passive probe (TPP0200) was used to monitor the capacitor voltage. The total input power, corresponding to the power drawn from the wall, was measured using a power analyzer (Extech Instruments, 380803). In each experiment, the input power was fixed, and plasma power was measured.

CO_2_ (bone-dry), H_2_ (UHP, 5.0), and Ar (UHP, 5.0) in a 1:3:1 ratio were introduced into the reactor at total flow rates of 20, 40, and 60 cm^3^ min^–1^. Residence times for 20, 40, and 60 cm^3^ min^–1^ are 7.53, 3.77, and 2.51 s, respectively, for the reactor volume of 2.5 cm^3^. The individual gas flows were regulated using three mass flow controllers (BROOKS 5850E) equipped with a BROOKS 0254 readout and set point controller, and the total flow rate was monitored using a mass flow meter (Omega FMA 1808A) and a soap bubble flow meter. The composition of the reactor effluent was analyzed using online gas chromatography (SRI 8610C) with thermal conductivity detection for CO_2_, CO, CH_4_, and Ar and flame ionization detection for hydrocarbon products. *X*_CO_2__, CO_2_ conversion, was calculated using eq 1, and *S*_*j*_, selectivity to product *j* (with *n*_*j*_ representing the number of carbons in species *j*) was calculated using eq 2.

$$X_{CO_{2}}=\frac{P_{CO_{2,in}}\left(\mathrm{kPa}\right)-P_{CO_{2,out}}\left(\mathrm{kPa}\right)}{P_{CO_{2,in}}\left(\mathrm{kPa}\right)}$$ 1

$$S_{j}=\frac{P_{j,out}{\left(\mathrm{kPa}\right)}^{*}n_{j}}{P_{CO_{2,in}}\left(\mathrm{kPa}\right)-P_{CO_{2,out}}\left(\mathrm{kPa}\right)}$$ 2

The specific energy input (SEI), which denotes the amount of energy supplied to the plasma per volume of gas entering the discharge, was calculated using eq 3.

$$SEI\left(\frac{kJ}{L}\right)=\left(\frac{Plasma\;power{\left(\frac{J}{s}\right)}^{*}60}{Total\;Flow\;Rate\left(\frac{mL}{\min}\right)}\right)$$ 3

Each experiment was performed independently in triplicate for uncertainty analysis. For each experiment, four individual data points were averaged, and error was taken as the standard deviation of the mean; this was performed for both the reactor performance data and for the power measurements. The three experiments were averaged, and error propagated accordingly and are shown as the error bars in all figures. Electrical characterization experiments discussed in Section 2.3 were also repeated three times, and data were treated as described.

The bulk gas temperature of the empty-DBD and packed-bed plasma reactor was estimated using a K-type thermocouple. Direct temperature measurements using an internal thermocouple aligned with the center of the plasma discharge zone during plasma operation are challenging due to electromagnetic interference, plasma–thermocouple interactions, and potential perturbation of the discharge. The plasma was operated at different input powers (20, 30, and 40 W) and a gas flow of 20 mL min^–1^ until steady-state operation was reached, defined by stabilization of the peak-to-peak discharge voltage at a constant value over time (approximately 1 h). Subsequently, the plasma power and gas flow were switched off simultaneously, and the thermocouple was immediately (within 10 s) inserted through the bottom of the reactor into the packed bed region to measure the residual gas temperature. This measurement was repeated independently three times for each bed and condition.

### 2.3 Electrical Characterization during Plasma-Enhanced CO_2_ Hydrogenation Reactions

Three waveforms were recorded with the oscilloscope to analyze the discharge behavior: (i) the applied voltage *V*(*t*), (ii) the voltage across a reference series capacitor *V*_c_(*t*) (*C*_ref_ = 10 nF), and (iii) the instantaneous current *I*(*t*) measured by a Pearson probe. Both *V*_c_(*t*) and *I*(*t*) are sinusoidal with numerous superimposed spikes (micro discharges) with very short rise times (<10 ns). The charge transferred through the reactor *Q­(t)* was calculated by the reference capacitor.

$$Q\left(t\right)=C_{ref}V_{c}\left(t\right)$$ 4

Plotting *Q* versus the reactor voltage *V* over one period yields the Lissajous figure. The plot area equals the energy dissipated per cycle; therefore, the plasma power *P* is given by eq 5, where *f* is frequency and *T* is the period,

P=f∮QVdV 5

where *f* = $\frac{1}{T}$ = 23.5 kHz.

For a DBD, the *Q*–*V* plot is well described by a parallelogram with two distinct regimes, as shown in Figure 2.^62^ In the nondischarging (capacitive) portions, represented by lines AB and CD, no plasma is present and the slope d*Q*/d*V* equals the cell capacitance *C*_cell_. Electrically, the reactor can be considered two capacitors in series: the dielectric barrier(s) and the gas/packed gap.

$$\frac{1}{C_{cell}}=\frac{1}{C_{diel}}+\frac{1}{C_{gap}}$$ 6

**Figure 2:**
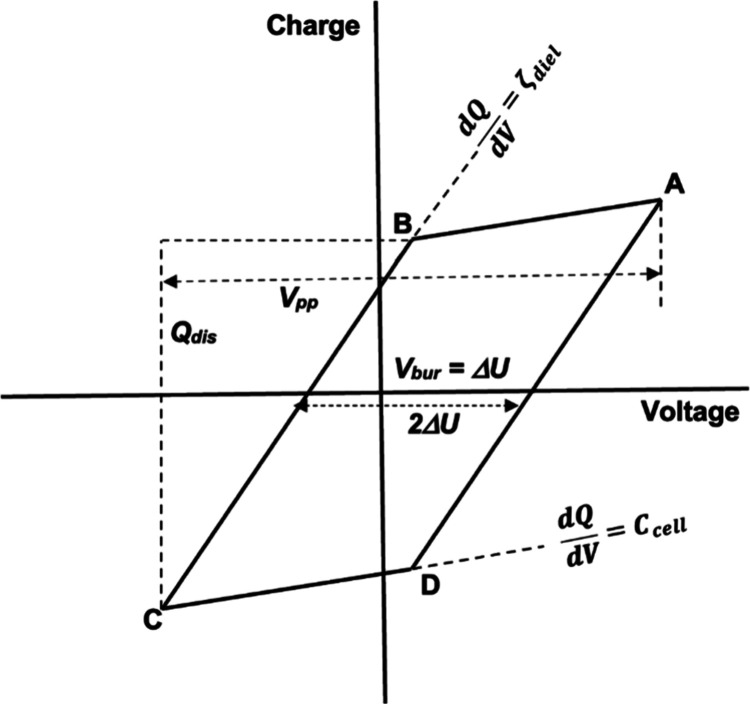
Annotated Lissajous figure showing the fitted linear model (parallelogram) as determined by a MATLAB script, with annotations indicating the information obtained from the plot including displacement charge (*Q*_dis_), peak-to-peak voltage (*V*_pp_), burning voltage (*V*_bur_ = Δ*U*), effective capacitances of the dielectric (ζ_diel_), and the cell capacitance (*C*_cell_).

*C*_cell_ was experimentally determined under plasma-off conditions using an LCR meter (BK Precision) equipped with a Kelvin clip (TL8KC1). Since direct capacitance measurement at the operating frequency of 23.5 kHz was not available, *C*_cell_ was measured over the accessible frequencies of the instrument (1, 10, and 100 kHz). The obtained values were therefore used to represent the static cell capacitance at the operating frequency. These plasma-off measurements provide an independent reference for comparison with capacitance values extracted from the nondischarging slope of the Lissajous *Q*–*V* plots during plasma operation.

The dielectric capacitance *C*_diel_ is set by the two concentric quartz tubes, for a coaxial geometry,

$$\frac{1}{C_{diel}}=\frac{1}{C_{inner}}+\frac{1}{C_{outer}}=\frac{\ln\left(\frac{r_{2,inner}}{r_{1,inner}}\right)+\ln\left(\frac{r_{2,outer}}{r_{1,outer}}\right)}{2\pi \varepsilon_{0}\varepsilon_{r}L}$$ 7

where *ε*_r_ = 4–7 for quartz^63^ and *L* is the axial length of the plasma discharge zone (0.05 m), *r*_1_ is the inner radius of the quartz tube, and *r*_2_ is the outer radius of the quartz tube, giving *C*_diel_ = 21 pF. When the gas-gap voltage exceeds the ignition voltage, the plot follows the discharge segments BC and DA. When charge transfers from one electrode to the opposite electrode during a discharge cycle, the slope in these segments equals *C*_diel_ (the gas gap no longer behaves capacitively). If the gap does not fully bridge, then the fitted slope is smaller and termed as the effective dielectric capacitance ζ_diel_ ≤ *C*_diel_. In that case, the Peeters–van de Sanden equivalent circuit (Figure 3) describes a partially discharging reactor with a nondischarging fraction *α* and discharging fraction *β* of the surface where *α* + *β* = 1.^64^

$$\alpha =\frac{C_{diel}-\zeta_{diel}}{C_{diel}-C_{cell}}$$ 8

$$\beta =\frac{\zeta_{diel}-C_{cell}}{C_{diel}-C_{cell}}$$ 9

**Figure 3:**
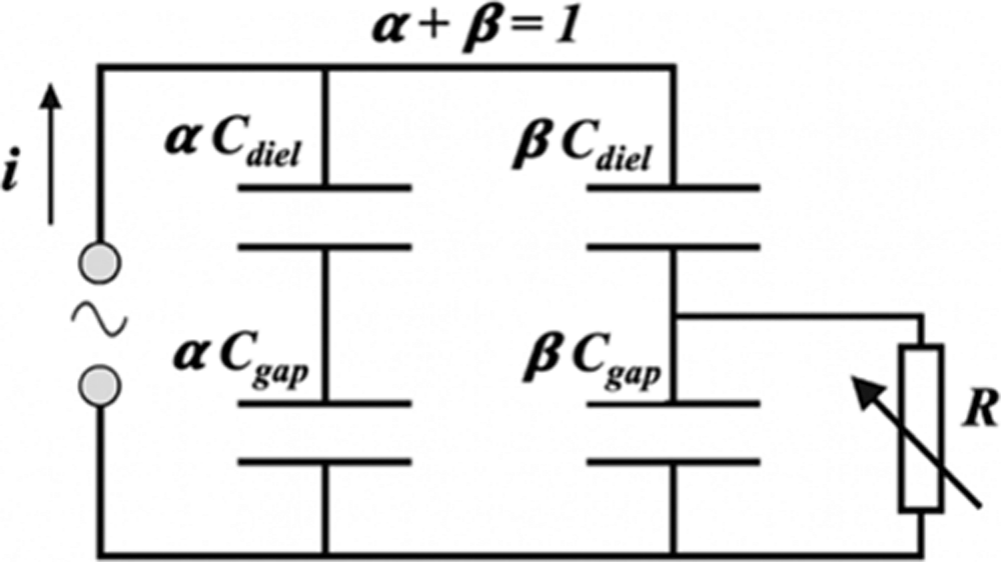
Equivalent circuit for partial discharge, adapted with permission from [^64^]. Copyright 2014 IOP Publishing Limited.

It has been reported that the interpretation of the current and voltage waveform for DBDs with variable capacitance *C*_diel_ is challenging, but the equivalent circuit for the partial discharge can be used for characterization of the discharge expansion over the dielectric surface. In packed-bed DBDs, each pellet acts as a local capacitor; the extent to which discharges propagate over pellet surfaces and thus the transition from capacitive to conducting behavior can be quantified by *α* and *β*.^36,65^ The burning voltage *V*_bur_ (voltage at which the discharge can sustain once ignited) where partial discharging occurring is calculated by eq 10.^36^ All the parameters mentioned here were calculated by fitting Lissajous plot to a parallelogram which was done automatically by using a MATLAB script.

$$V_{bur}=\frac{1-\frac{C_{cell}}{C_{diel}}}{1-\frac{C_{cell}}{\zeta_{diel}}}\Delta U$$ 10

The instantaneous current profiles measured by the Pearson probe were used to determine the number of micro discharges (*N*_md_) occurring within the plasma region and to compare discharge behavior under different conditions. Owing to the reactor’s low inductance, the instantaneous current captures the fast-varying total discharge current, which consists of the plasma current (conduction current) and the dielectric current (capacitive current).^66^ The plasma current appears as a series of sharp, superimposed pulses (micro discharges) on the capacitive waveform. A MATLAB script was employed to identify individual micro discharges by fitting the dielectric current as the baseline and applying height and width threshold values to remove signal noise, resonance, and systematic measurement errors.^65–67^ The accuracy of this approach was validated by comparing the calculated *N*_md_ values with manually counted peaks.

### 2.4 In Situ Plasma Imaging Using High-Speed Photography

A custom-built chamber was designed to characterize plasma discharge behavior of CeO_2_, as shown in Figure 4. The chamber consists of a hollow octagon housing a quartz tube of comparable thickness to the inner quartz tube used in the DBD experimental setup. Pellets of the CeO_2_ were affixed to Kapton tape on the base of the octagon using silicone RTV. A high-voltage Cu electrode was positioned inside the quartz tube, while the ground connection was placed at the base of the octagon, covered by a quartz disk of comparable thickness to the outer quartz tube. Although geometric differences exist between the reactor and characterization chamber, comparable near-surface plasma discharges are expected owing to the same radius of the high-voltage electrode and quartz dielectric. However, the applied voltages in the characterization chamber are not directly comparable to those in the DBD reactor and are used solely for qualitative assessment of near-surface discharge behavior. Prior to video recording, the chamber was first evacuated by a vacuum pump (Agilent Technologies IDP2). Subsequently, it was purged with 5.0 ultrahigh-purity gases of CO_2_, H_2_, and Ar in a 1:3:1 ratio with a total flow rate of 20 cm^3^ min^–1^ and pressure was stabilized at atmospheric pressure. The individual gas flows were regulated using three mass flow controllers (MKS 1480A-25078) equipped with an MKS 647B readout and set point controller and monitored using a mass flow meter (Alicat Scientific MC) and a soap bubble flow meter. Then, a voltage ranging from 18 to 40 kV was applied. The resulting discharge behavior was recorded using a Phantom TMX 7510 high-speed camera, aligned with the chamber window. Videos were captured at a resolution of 1280 × 760 pixels, with a frame rate of 300 fps, and an exposure time of 3300 μs by using a 25 mm F2.8 Ultra Macro 2.5–5.0X lens (Laowa).

**Figure 4:**
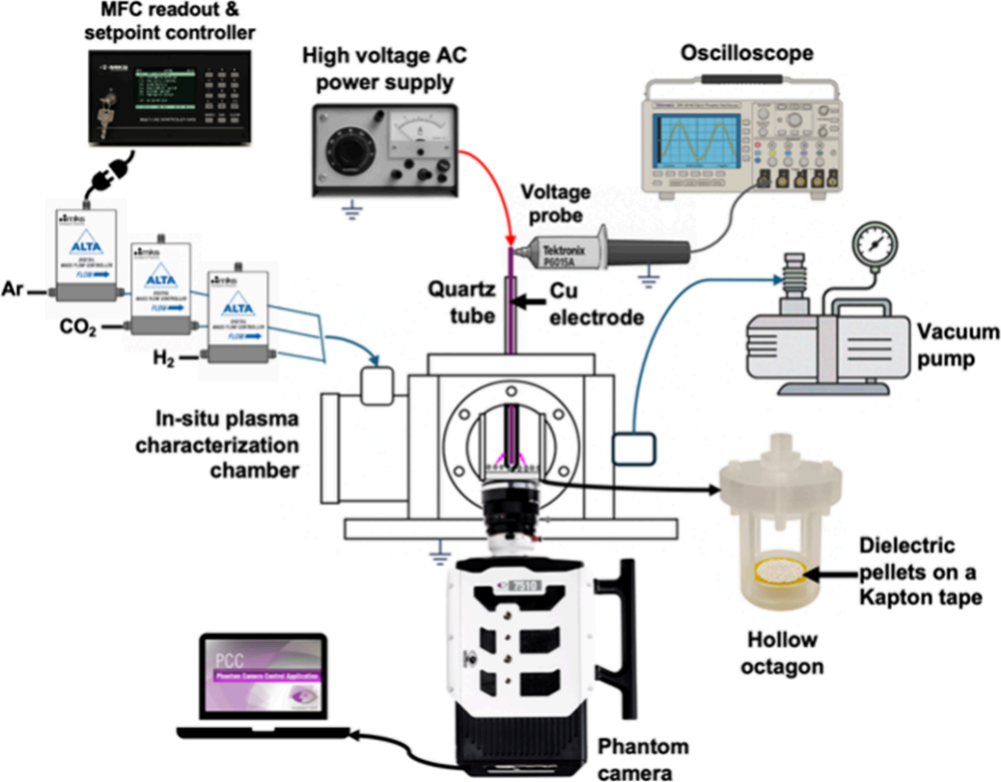
Schematic of the optical chamber setup for plasma discharge behavior visualization. Gas flow paths are indicated by blue arrows, high-voltage electrical connection to the reactor is shown in red arrow, and gray arrows denote the measurements of electrical signals.

## 3 Results and Discussion

### 3.1 CO_2_ Hydrogenation over DBD Packed Beds of Different Oxides

#### 3.1.1 Reactor Performance in an Empty DBD Reactor

This section presents the performance of the DBD reactor alone, evaluated by CO_2_ conversion and CH_4_ selectivity at varying specific energy inputs (SEI; eq 3). Here, we refer to two powers: the input power and the discharge (plasma) power obtained from Lissajous plots, as described in Section 2. As shown in Figure 5, CO_2_ conversion increases with SEI, which was varied by changing the input power and residence time (7.53–2.51 s) for the empty reactor. Plasma (discharge) power and steady-state voltage remained consistent across all residence times at different input powers (Figures S1 and S2). CO_2_ conversion increases monotonically with increasing SEI values, irrespective of whether SEI is altered by varying plasma power or reactor residence times. This is consistent with literature observations.^65^ SEI reflects the electrical energy deposited per volume of reactants (eq 3). At a given residence time, increasing plasma power primarily increases the density of energetic electrons while not influencing their average temperature, which increases the probability of electron-molecule (with CO_2_, H_2_, and Ar) collisions. These collisions drive Ar excitation/ionization, CO_2_ dissociation to CO, and H_2_ activation, which together influence the conversion of CO_2_.^16,68,69^ The predominant product of CO_2_ conversion under these conditions is CO (selectivities >∼90%). At low CO_2_ conversions, only CO is produced in measurable pressures in the reactor effluent. Small amounts of CH_4_ are detected, with selectivities reaching up to 8% at higher SEI values and, accordingly, greater CO_2_ conversions (Figure S3). These trends reflect the synergistic influence of plasma power and residence time: increasing plasma power may increase the density of energetic electrons, which in turn increase the frequency of CO_2_ dissociation events and enable further fragmentation of primary product CO into C^•^ and O^•^ radicals along with C^•^ hydrogenation reactions.^68^ The absence of coke deposition on the quartz dielectric walls during the experiments suggests that the generated C^•^ species were rapidly hydrogenated rather than accumulating as coke. Together, the combination of elevated plasma power and sufficient residence time promotes more extensive secondary reaction pathways, favoring CH_4_ formation.

**Figure 5:**
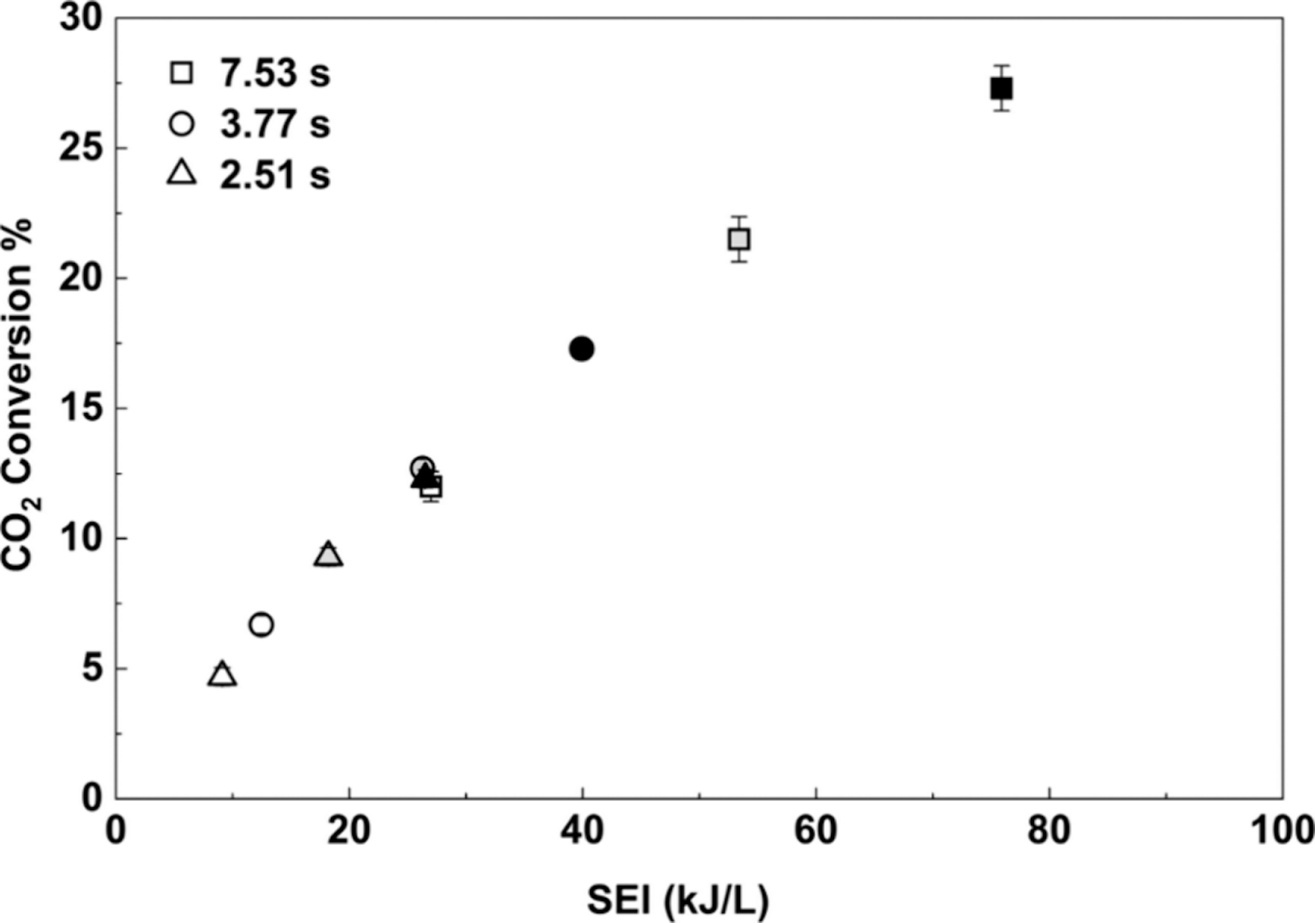
CO_2_ conversion as a function of SEI for an empty DBD reactor (CO_2_:H_2_:Ar = 1:3:1, frequency = 23.5 kHz, 1 atm, 298 K). Squares, circles, and triangles represent residence times of 7.53, 3.77, and 2.51 s, and white, gray, and black represent input powers of 20, 30, and 40 W, respectively.

#### 3.1.2 Electrical Characterization of the Empty-DBD Reactor

This section describes discharge behavior in a DBD reactor alone. The discharge behavior was analyzed by fitted Lissajous figures and instantaneous current profiles. Figure 6 presents the Lissajous figures for varying input powers of 20, 30, and 40 W for the empty DBD reactor at a residence time of 7.53 s. The calculated areas of these plots correspond to plasma powers of 9, 18, and 26 W, respectively. The electrical characteristics derived from the fitted Lissajous figures and instantaneous current profiles (Figure 7) are summarized in Table 1. At the same input power, varying the residence time produced no noticeable change in plasma power (Figure S2 and Table S1) or in the number of microdischarges, represented by the superimposed current peaks. Higher input power increases the Lissajous plot area, width (peak-to-peak voltage, *V*_pp_), slope of the discharge phase (effective capacitance, ζ_diel_), and height (displaced charge, *Q*_dis_) whereas the slope of the capacitive phase (cell capacitance, *C*_cell_) and the corrected burning voltage *V*_bur_ remains unchanged. This increased plasma power resulted in higher CO_2_ conversion as mentioned in the previous section.

**Figure 6:**
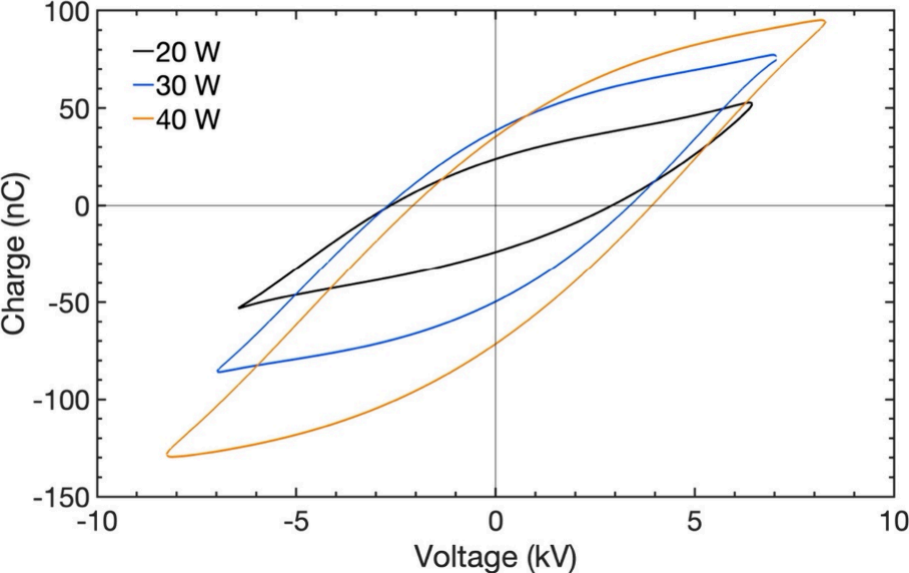
Lissajous plots at different input powers of 20, 30, and 40 W for an empty-DBD reactor at a residence time of 7.53 s (CO_2_:H_2_ Ar = 1:3:1, frequency = 23.5 kHz, 1 atm, 298 K).

**Table 1:** Calculated Discharge Characteristics Obtained from the Instantaneous Current Profiles and Fitted Lissajous Figures for the Empty DBD Reactor at Input Powers of 20, 30, and 40 W at a Residence Time of 7.53 s

Input power (W)	Plasma power (W)	*V*_pp_ (kV)	ζ_diel_ (pF)	*C*_cell_ (pF)	α	*V*_bur_ (kV)	*Q*_dis_ (nC)	*N*_md_
20	9.0 ± 0.1	12.6 ± 0.1	12.7 ± 0.1	4.4 ± 0.4	0.5	3.1	71 ± 2	21 ± 2
30	17.7 ± 0.3	14.2 ± 0.2	19.1 ± 0.4	3.9 ± 0.3	0.1	3.1	128 ± 3	40 ± 1
40	25.3 ± 0.3	16.4 ± 0.2	20.8 ± 0.2	4.3 ± 0.2	0.0	3	175 ± 1	38 ± 1

**Figure 7:**
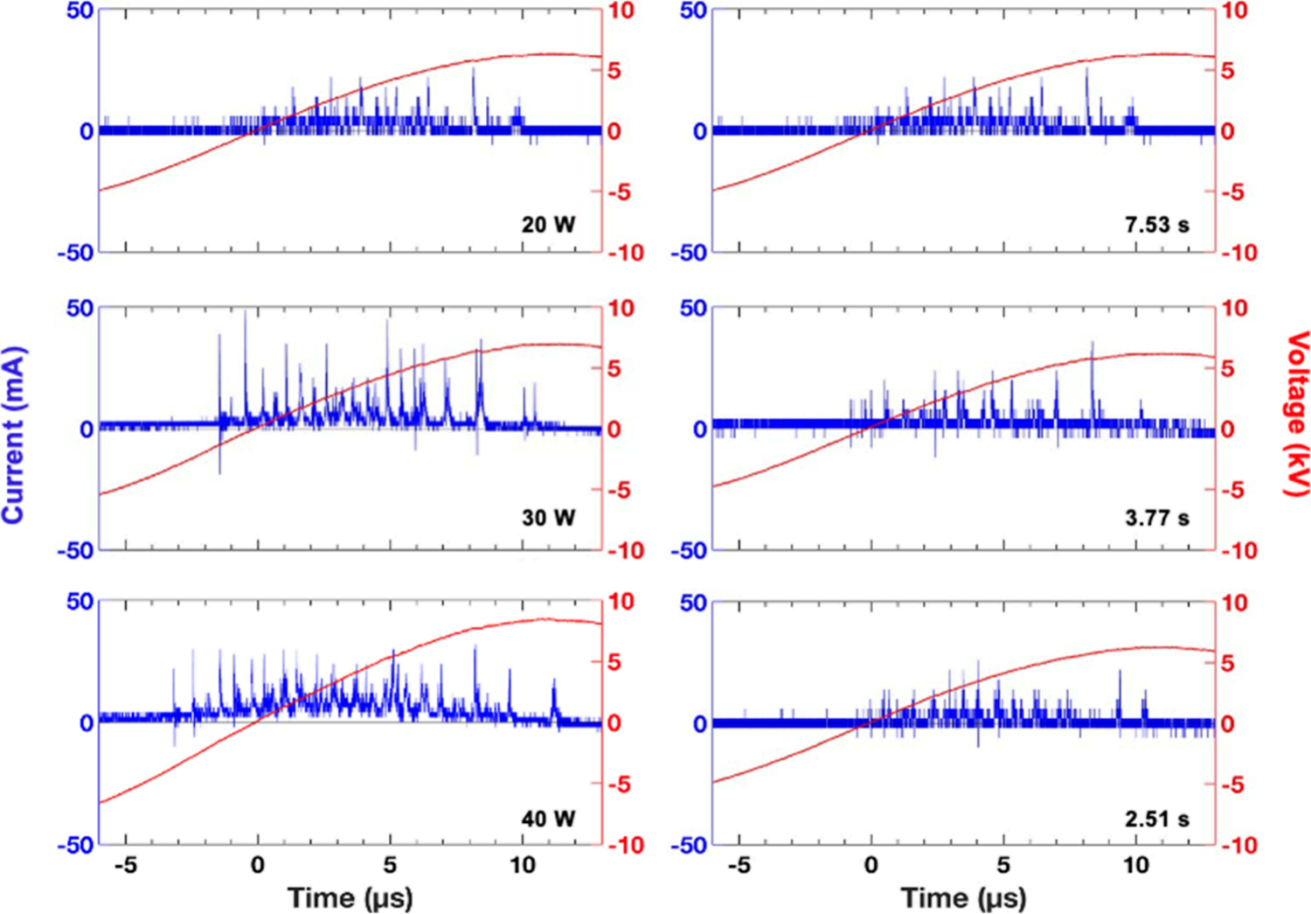
Instantaneous current profiles during the positive half-cycle of the applied voltage for the empty DBD reactor for different input powers at a residence time of 7.53 s (left) and for different residence times at an input power of 20 W (right).

The cell capacitance (*C*_cell_) remains similar across input powers because it depends only on the reactor’s geometry and dielectric materials, which were unchanged. In contrast, the effective capacitance (ζ_diel_) increases with input power due to alterations in the charge distribution, as discussed below.

Notably, at an input power of 20 W, the discharge was partial because ζ_diel_ < *C*_diel_ (21 pF), indicating that the residual charge stored on the dielectric surface (quartz) could not be completely transferred to the opposite electrode within each discharge.^36^ This partial discharging (*α* = 0.5, where 0 ≤ *α* ≤ 1) reflects the limited plasma formation and reduced reactor performance. Thus, it is essential to evaluate catalytic activity in plasma-catalytic experiments only when the reactor is fully discharging or when the extent of partial discharging is quantified. As input power increased, ζ_diel_ values also increased, and *α* → 0 at approximately 40 W, indicating full discharge operation. This corresponds to an applied voltage of ∼16 kV required to achieve optimal performance under fully discharging conditions for the empty-DBD reactor. This is further supported by the increasing *Q*_dis_ (the capacitive charge transferred through the dielectric barrier) by approximately 100 nC, along with the doubling of *N*_md_ as the input power increased from 20 to 40 W.

Although *N*_md_ remained similar at input powers of 30 and 40 W, the current profiles differed significantly. At 30 W, the discharges are distributed relatively evenly across the three peak-height bins, with a slightly greater number of weak (10–15 mA) discharges compared to moderate (15–25 mA) and strong (>25 mA), respectively (Figure S4). This distribution indicates a more diverse microdischarge population, including a substantial number of strong events. In contrast, at 40 W, the discharge population becomes skewed toward the two lower-intensity bins (10–15 and 15–25 mA), while the number of strong events *N*_md_ (>25 mA) decreases to roughly half of that observed at 30 W. This discharge behavior indicates a redistribution of discharge energy and altered plasma propagation dynamics at higher power. The higher-voltage amplitude and electric field at 40 W lead to greater surface charge accumulation on the dielectric, which modifies the local electric field suppressing the strong, localized discharges observed at 30 W. Consequently, subsequent micro discharges become weaker but more uniformly distributed, producing more medium-intensity peaks instead of fewer high-intensity ones.^52,70,71^ Thus, in DBD analysis, the intensity of a micro discharge (height of the current spike) correlates with the instantaneous discharge current (electron density), the extent of plasma channel propagation, and the energy dissipated per filament. So, when intensity decreases but the number of discharges remains similar, it indicates the discharge transitions from strong, localized microfilaments (more energetic, short-lived) to more diffuse, overlapping discharges (less energetic, longer lasting). This trend is further promoted by the use of a double dielectric barrier configuration, which enhances charge accumulation symmetry on both surfaces and suppresses localized field intensification, thereby generating a more uniform and stable discharge compared to reactors with a single quartz barrier.^72^

*V*_bur_ is expected to remain nearly constant as a function of *V*_pp_ in a fixed DBD geometry once gas breakdown occurs, as it represents the sustaining voltage required to keep the plasma micro discharges active after ignition. While earlier studies have reported a decrease in *V*_bur_ with increasing applied voltage, our results show similar *V*_bur_ even after applying the correction proposed by Butterworth et al. (∼3 kV), during the operating conditions (as *V*_pp_ increases from 12.5 to 16.5 kV).^36^

#### 3.1.3 Reactor Performance over DBD Packed Beds of Varied Oxides

Figure 8 shows CO_2_ conversion as a function of SEI in a DBD reactor packed with different dielectric constant materials (600–1000 μm aggregates): Al_2_O_3_ (ε_r_ = 9), CeO_2_ (ε_r_ = 25), TiO_2_ (ε_r_ = 80), and BaTiO_3_ (ε_r_ = 7000). CO_2_ conversion increases monotonically with SEI for a given oxide, but only Al_2_O_3_ reaches high conversion: 42% at SEI ∼71 kJ L^–1^ compared to the empty-DBD (27% at SEI ∼76 kJ L^–1^). In contrast, CO_2_ conversion over CeO_2_, TiO_2_, and BaTiO_3_ each remains below 10% even at higher SEI values, and product concentrations are below detection limits in the reactor effluent for SEI values <21 kJ L^–1^. The overall trend in CO_2_ conversion is Al_2_O_3_ > empty-DBD > BaTiO_3_ > TiO_2_ > CeO_2_, consistent with prior studies on CO_2_ dissociation over Al_2_O_3_ and BaTiO_3_ packed beds.^36^

**Figure 8:**
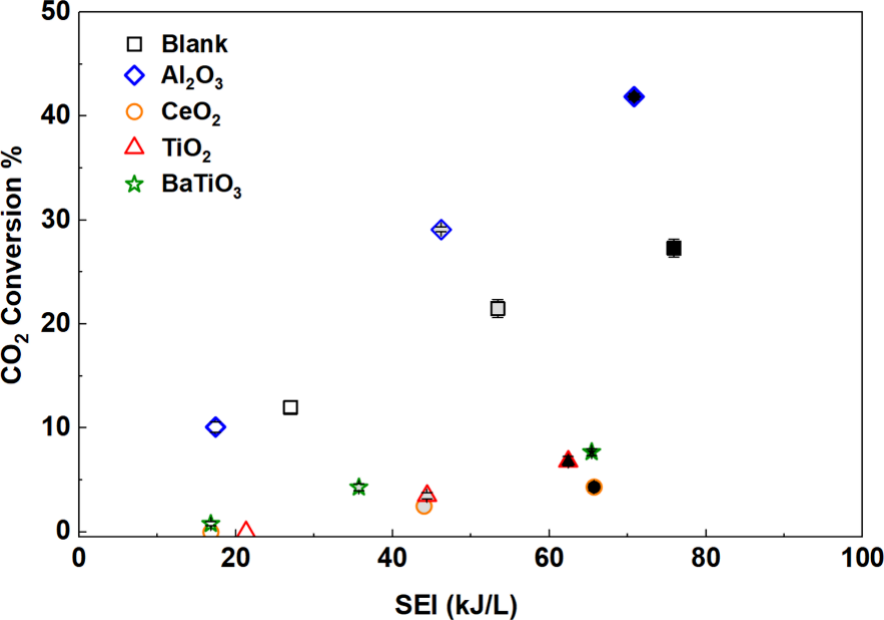
CO_2_ conversion as a function of SEI for the empty DBD reactor (black square) and reactor packed with Al_2_O_3_ (blue diamond), CeO_2_ (orange circle), TiO_2_ (red triangle), and BaTiO_3_ (green star) at different input powers (white, gray, and black represent input powers of 20, 30, and 40 W, respectively) (CO_2_:H_2_:Ar = 1:3:1, feed = 20 mL min^–1^, frequency = 23.5 kHz, particle size = 600–1000 μm, 1 atm, 298 K).

These trends in CO_2_ conversion could result from differences in surface area or porosity that influence the formation of microdischarges. The measured BET surface area, pore volume, and average pore size of all oxides are summarized in Table S2. Among the investigated dielectric materials, Al_2_O_3_ exhibits the highest surface area and pore volume (146 m^2^ g^–1^ and 1.06 cm^3^ g^–1^, respectively), indicating a larger accessible internal surface compared to the other materials. However, pores contained in these materials are smaller than the Debye length, meaning such internal pores (average pore size 10.7–24.7 nm) are not expected to directly play a role. Nevertheless, streamers may partially interact with pore openings, and plasma-generated reactive species may diffuse into internal surfaces. Previous modeling studies^51,73,74^ have examined these effects and suggest their influence plays a secondary role compared to that of the dielectric packing on the overall discharge behavior.

The reactor porosity and residence time differ among materials (Table S2), and measured plasma power also varies with packing material (Figure S5). Consequently, each bed operates at a different SEI value. A longer residence time would be expected to increase CO_2_ conversion by increasing the time for the gas to dwell in the discharge zone. Residence times are greatest in Al_2_O_3_ beds, followed by those of CeO_2_, TiO_2_, and BaTiO_3_ (Table S2). CO_2_ conversion, however, only weakly correlates with these residence times (Figure 8). Instead, all high-*ε*_r_ materials exhibit a low CO_2_-conversion-to-SEI ratio. These trends suggest that CO_2_ conversion is a stronger function of discharge localization than of gas residence time.

We also examine the possible existence of temperature differences among packed beds and their influence on observed reactivity. With increasing input power, the energy density within the plasma increases, resulting in more frequent and intense micro discharges and greater local heating; these temperature differences could influence reactor performance via thermal contributions. For a given reactor packing, measured temperatures are greatest for the highest input power (40 W) and decrease monotonically with decreasing input power (Figure S6; 413–538 K for empty reactors and 378–498 K for packed beds). These temperatures are in good agreement with those reported for atmospheric-pressure DBD plasma reactors in the literature.^75–77^ CO_2_ conversion, however, is not correlated with these bulk gas temperatures (Figure 8), as would be expected for reactions where thermal effects dominate; empty reactors have the highest temperature at all conditions (Figure S6), but Al_2_O_3_ beds show highest CO_2_ conversion. Previous work has shown that CO_2_ conversion is nearly independent of temperature in these ranges, either varied through external heating^78^ or by cooling with water.^77^ Taken together, these data suggest that temperature differences play a minor role in these packed beds.

These differences reflect distinct discharge topologies imposed by dielectric properties. With Al_2_O_3_ (low *ε*_r_), the onset voltage for streamer formation is lower, leading to a direct transition into surface streamers that propagate through the void channels. Polarization at particle contacts sharpens local fields, enabling efficient electron–molecule activation in the bulk gap, despite a lower measured plasma power (and thus lower SEI) than the empty reactor. For high *ε*_r_ packings (BaTiO_3_, TiO_2_, and CeO_2_), discharges preferentially anchor at pellet–electrode or pellet–pellet contact points, producing more intense but highly localized partial discharges.^42,53^ Literature reports indicate that, at high input power, these effects are amplified: stronger polarization at contact points increases local electric fields, which accelerates electrons toward the dielectric surfaces, enhancing electron loss (charge scattering) and dielectric suppression.^52,79^ This reduces the effective discharge volume, limits the extent of plasma–oxide interaction, and decreases overall CO_2_ conversion. Among the high-*ε*_r_ materials, BaTiO_3_ exhibits slightly higher conversion, consistent with the presence of more intense localized streamer discharges compared to TiO_2_ and CeO_2_, but overall, all high-*ε*_r_ materials show low and similar conversion due to these effects.

Figure S7 shows the relationship between CH_4_ selectivity and CO_2_ conversion for the packed beds of different oxides. CH_4_ selectivity increases with conversion but remains below 10% for all packed-bed materials. For Al_2_O_3_, the CH_4_ selectivity increases to ∼10% as the CO_2_ conversion rises from 10 to 42%. In contrast, for BaTiO_3_, the CH_4_ selectivity increases to 6% at a lower CO_2_ conversion of 8%. Only Al_2_O_3_ and BaTiO_3_ produce higher hydrocarbons (C_2+_) among the packed beds. However, Al_2_O_3_ yields only ∼1% total C_2+_ selectivity: mainly ethane and propane, each accounting for about 5% of the hydrocarbon fraction (including methane) at 42% CO_2_ conversion. In comparison, BaTiO_3_ produces ∼2% total C_2+_ selectivity, distributed across several C_2+_ including methanol, ethane, ethene, propane, propene, butane, and butene, with a combined hydrocarbon selectivity of ∼20% (including methane). These differences may reflect distinct discharge characteristics governed by the dielectric properties of the packing materials. In Al_2_O_3_ and the empty-DBD reactor, void-spanning surface streamers promote CO_2_ dissociation primarily to CO, with limited secondary hydrogenation. In contrast, the contact-anchored partial discharges formed in BaTiO_3_ produce more intense but spatially localized plasma regions, favoring secondary hydrogenation pathways even at low CO_2_ conversion.

Literature reports that BaTiO_3_ packed beds, in particular, generate substantially higher mean electron energies compared to both the empty DBD and Al_2_O_3_ beds, resulting in the formation of more chemically reactive CH_*x*_^•^ species.^52,68,80^ We surmise that such phenomena underpin secondary methane selectivities here, in the absence of catalytic metal sites.

Overall, the dielectric constant governs discharge mode and energy coupling, which in turn controls the balance between dissociation-dominated and secondary hydrogenation regimes. Low-*ε*_r_ materials, such as Al_2_O_3_, promote more uniform discharges and efficient CO_2_ dissociation, resulting in higher overall conversion. In contrast, high-*ε*_r_ materials, such as BaTiO_3_, generate more localized and intense discharges that favor secondary hydrogenation pathways but yield lower overall CO_2_ conversion.

#### 3.1.4 Electrical Characterization of DBD Packed Beds of Varied Oxides

Here, we describe the discharge behavior with the packed bed DBD reactor. Discharge behavior was analyzed by Lissajous figures and instantaneous current profiles. Figure 9 and Table 2 summarize the electrical characteristics of the empty DBD and for packed-bed configurations with Al_2_O_3_ (*ε*_r_ = 9), CeO_2_ (*ε*_r_ = 25), TiO_2_ (*ε*_r_ = 80), and BaTiO_3_ (*ε*_r_ = 7000) at an input power of 20 W using particle sizes 600–1000 μm. The introduction of dielectric pellets substantially modifies the reactor’s electrical characteristics, as evidenced by variations in the plot areas (plasma power), slopes (capacitance), heights (charge), and widths (voltage) of the Lissajous figures. The empty DBD exhibits the highest plasma power of 9 W, corresponding to the largest Lissajous plot area. In contrast, all packed-bed configurations show similar, lower plasma power values of around 6 W. The introduction of packing materials into the annular DBD plasma region thus reduces the plasma power by about 3 W while increasing the applied voltage by approximately 2 kV.

**Figure 9:**
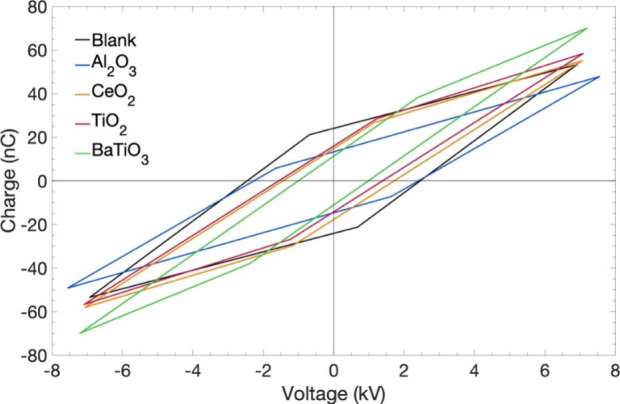
Fitted Lissajous parallelograms for empty-DBD, and packed beds of Al_2_O_3_, CeO_2_, TiO_2_, and BaTiO_3_ (CO_2_:H_2_:Ar = 1:3:1, input power = 20 W, feed = 20 mL min^–1^, particle size = 600–1000 μm, frequency = 23.5 kHz, 1 atm, 298 K).

**Table 2:** Calculated Discharge Characteristics Obtained from the Instantaneous Current Profiles and Fitted Lissajous Figures for the Empty DBD Reactor and Reactor Packed with Al_2_O_3_, CeO_2_, TiO_2_, and BaTiO_3_ at Input Power of 20 W and Total Flow Rate of 20 cm^3^ min^–1^

Dielectric material	Plasma power (W)	*V*_pp_ (kV)	ζ_diel_ (pF)	*C*_cell_ (pF)	*α*	*V*_bur_ (kV)	*Q*_dis_ (nC)	*N*_md_
	9.0 ± 0.1	12.6 ± 0.1	12.7 ± 0.1	4.4 ± 0.4	0.5	3.1	71 ± 2	21 ± 2
Al_2_O_3_	6.0 ± 0.4	14.4 ± 0.3	9.1 ± 0.2	4.5 ± 0.2	0.7	3.7	49 ± 0	9 ± 0
CeO_2_	5.9 ± 0.3	14.5 ± 0.4	9.6 ± 0.4	4.7 ± 0.4	0.7	2.3	84 ± 0	20 ± 1
TiO_2_	6.2 ± 0.4	14.1 ± 0.6	10.3 ± 0.1	5.1 ± 0.2	0.7	2.2	85 ± 2	18 ± 0
BaTiO_3_	5.8 ± 0.3	14.7 ± 0.5	11.0 ± 0.4	6.0 ± 0.6	0.7	1.5	105 ± 2	29 ± 1

This behavior indicates that the presence of packing requires a higher applied voltage to sustain the discharge at the same input power. The addition of dielectric pellets increases the effective dielectric constant of the discharge region because part of the gas volume is replaced by solid dielectric. As a result, the electric field distribution is altered: a larger portion of the applied voltage drops across the dielectric particles rather than the gas phase, reducing the electric field strength in the gas voids where plasma formation occurs.^79^ To initiate and sustain the plasma discharge, a higher applied voltage is therefore required to provide a sufficient local electric field. The decrease in plasma power arises because the packing decreases the effective discharge volume and discharge current, leading to lower overall energy dissipation within the plasma. Similar trends have been reported in previous studies.^79,81,82^ This effect becomes more pronounced with increasing input power. As the input power increases, the empty DBD reactor continues to deliver the highest plasma power of 18 and 25 W at input powers of 30 and 40 W, respectively, whereas the plasma power progressively decreases with increasing dielectric constant at the same input powers (see Tables S3 and S4). As shown in Figure S5, among the packed beds, Al_2_O_3_ exhibits the highest plasma power while BaTiO_3_ delivers the lowest, decreasing from 15.4 to 11.7 W and 23.2 to 20.5 W at 30 and 40 W (input power), respectively. *V*_pp_ also increases with input power, with Al_2_O_3_ showing an increase of ∼1 kV, whereas high-ε_r_ materials show a larger increase of about 3 kV. The trends in ζ_diel_, *C*_cell_, *Q*_dis_, and *N*_md_ as a function of SEI further corroborate this behavior (Figure 10).

**Figure 10:**
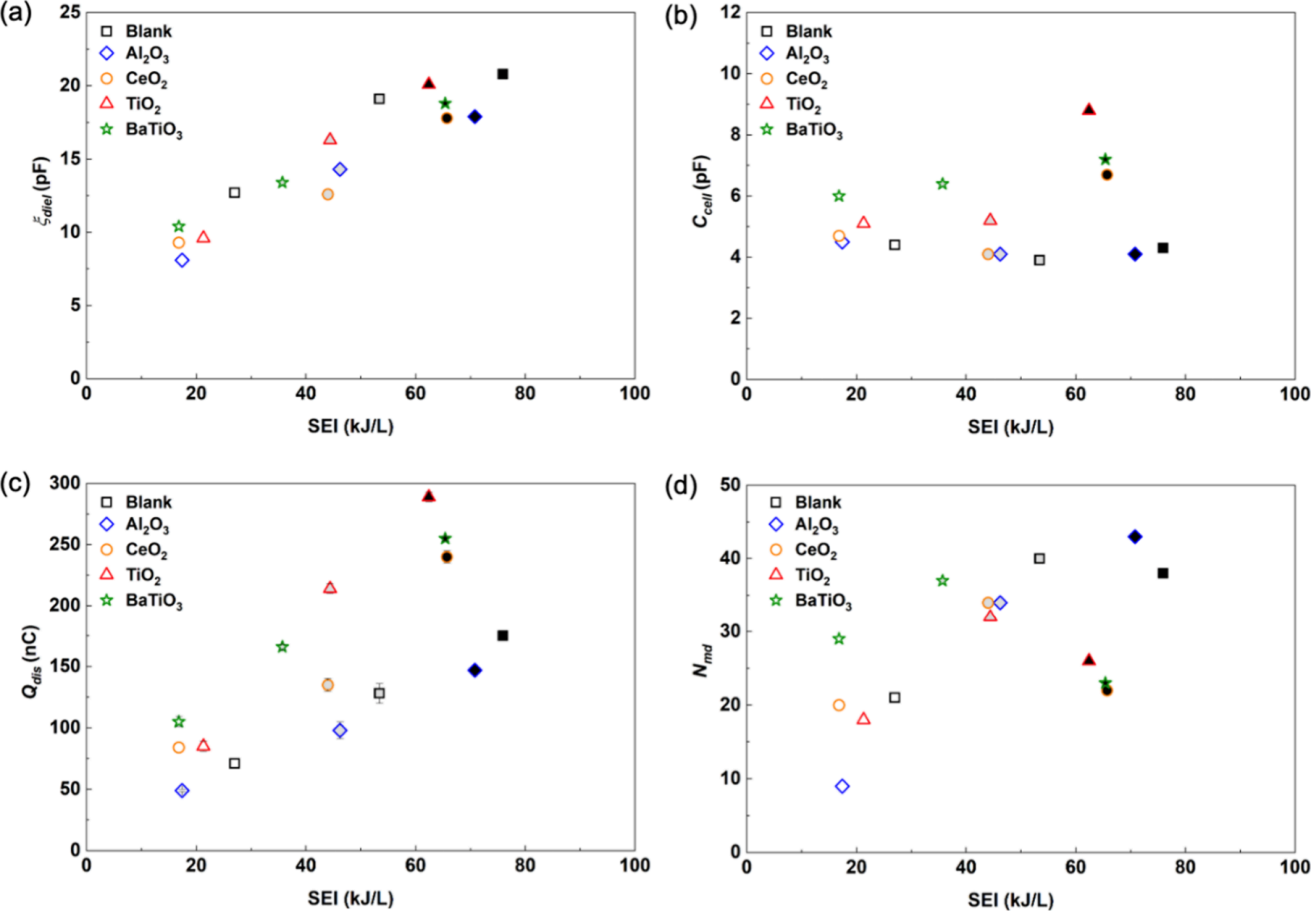
Calculated (a) ζ_diel_, (b) *C*_cell_, (c) *Q*_dis_, and (d) *N*_md_ obtained from the fitted Lissajous figures and instantaneous current profiles as a function of SEI for the empty DBD reactor (black square) and reactor packed with Al_2_O_3_ (blue diamond), CeO_2_ (orange circle), TiO_2_ (red triangle), and BaTiO_3_ (green star) at different input powers (white, gray, and black represent input powers of 20, 30, and 40 W, respectively) and total flow rate of 20 cm^3^ min^–1^.

In a packed bed reactor, α represents the fraction of the stored dielectric charge that reaches the opposing electrode during a discharge cycle.^36^ At a low input power of 20 W, α values of 0.5 for the empty-DBD reactor indicate partial charge transfer, as discussed in Section 3.1.2. In contrast, all packed beds exhibit *α* = 0.7, signifying more limited charge transfer compared to the empty-DBD reactor. This behavior correlates with the zero CO_2_ conversion observed for the high-*ε*_r_ materials and the comparable conversion achieved with Al_2_O_3_, consistent with the distinct discharge behaviors described in Section 3.1.3 (Figure 8).

Both ζ_diel_ and *C*_cell_ increase proportionally with increasing dielectric constant, leading to similar *α* values across all input powers. As the input power increases, charge transfer becomes more complete (i.e., *α* → 0), and the intrinsic dielectric properties of the packing material increasingly dominates the discharge characteristics. At all input powers, *C*_cell_ increases with the dielectric constant, indicating a stronger capacitive contribution from the packed bed, which stores charge locally but limits complete transfer across the discharge gap. For BaTiO_3_, each pellet acts as an individual capacitor that stores more dielectric charge instead of transferring it. The result is localized field enhancement and restricted discharge propagation, confined primarily to pellet–pellet or pellet–electrode contacts points rather than spanning across the voids. In contrast, Al_2_O_3_ exhibits a *C*_cell_ value nearly equal to that of the empty-DBD reactor (Figure 10), as supported by plasma-off LCR measurements (Figure S8), suggesting that the discharge continues to propagate effectively through the interparticle void channels, maintaining plasma activity throughout the discharge region. The plasma-off LCR measurements represent the static capacitance of the fully polarized reactor and packed bed, whereas the capacitance extracted from the nondischarging slope of the Lissajous figures reflects an effective cell capacitance under partial discharge conditions, which may remain lower than the static value due to incomplete dielectric polarization. Consequently, *Q*_dis_ is highest for the high-*ε*_r_ materials, as it increases with *C*_cell_ and is lowest for Al_2_O_3_. The lower *Q*_dis_ observed for Al_2_O_3_ arises from its low dielectric constant, which provides insufficient polarization to compensate for the reduced gas-phase discharge volume, leading to a smaller overall *Q*_dis_ compared to the empty DBD reactor. A similar trend is reflected in *V*_bur_, which decreases for BaTiO_3_ due to its high *ε*_r_ and strong polarization that enhances the local electric field, allowing plasma to ignite and sustain at lower voltage. In contrast, Al_2_O_3_, with weaker polarization and decreased effective gas discharge volume, requires a higher voltage to sustain the plasma. This behavior was also reported in previous studies.^36,79^

At the same time, *N*_md_ exhibits an opposite trend. At an input power of 20 W, *N*_md_ is significantly lower for Al_2_O_3_ than for the other packed beds and the empty DBD reactor. However, as the input power increases and *α* → 0, *N*_md_ for Al_2_O_3_ rises monotonically and eventually surpasses that of all other configurations (Figure 11 and Figures S9 and S10). For the high-*ε*_r_ materials, *N*_md_ initially increases with input power but subsequently decreases at higher power input. Literature reports indicate that materials with higher dielectric constants typically exhibit the lowest *N*_md_ values, as they tend to form localized discharges.^36,52,67^ This occurs because such materials store charge rather than transferring it, meaning their characteristic dielectric behavior becomes evident only at higher input powers or under full discharge conditions.

**Figure 11:**
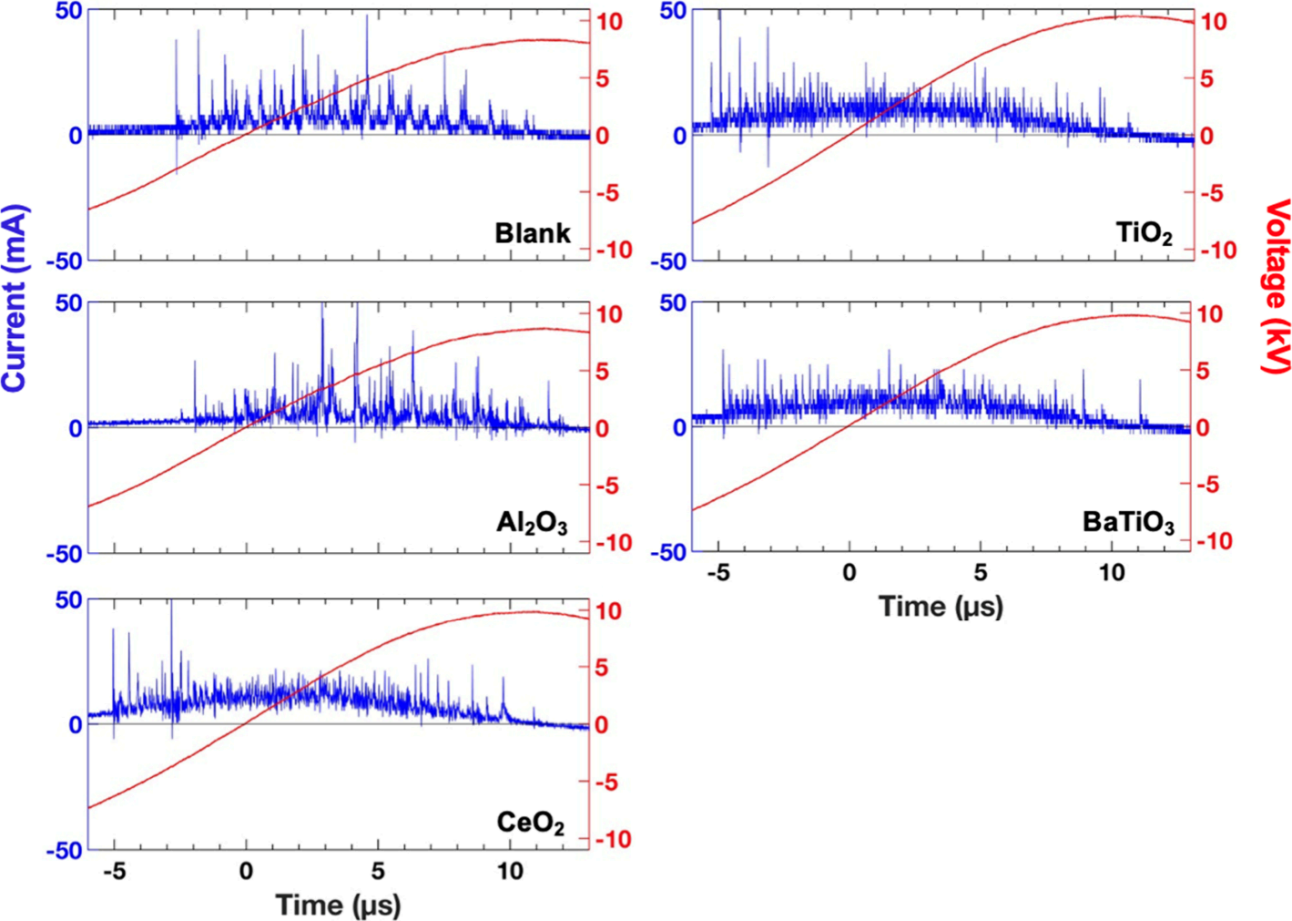
Instantaneous current profiles during the positive half-cycle of the applied voltage for the empty DBD reactor and packed-bed configurations of Al_2_O_3_, CeO_2_, TiO_2_, and BaTiO_3_ at an input power of 40 W.

Both the empty DBD and Al_2_O_3_-packed reactor display numerous high-intensity micro discharge peaks, signifying extended plasma channel propagation, whereas the high-*ε*_r_ materials exhibit fewer and weaker peaks, consistent with earlier studies.^36,42,52,67,79^ The enhanced micro discharge activity observed for Al_2_O_3_ at elevated powers likely contributes to its higher CO_2_ conversion efficiency relative to the other packed beds. Therefore, to enable a proper comparison of catalytic activity among different dielectric materials, it is essential to operate the DBD reactor under fully developed discharge conditions.

### 3.2 CeO_2_ Packed Beds: Effect of Particle Size on Reactor Performance and Electrical Characteristics

Figure 12 shows CO_2_ conversion as a function of SEI, in a DBD reactor packed with CeO_2_ pellets spanning four size ranges (180–250, 350–500, 600–1000, and 1400–1700 μm) at a residence time of ∼6 s. It can be noted that the reactor porosity of the various sizes of CeO_2_ pellets and residence times are quite similar as shown in Table S5, but plasma power changes (Figure S11). CO_2_ conversion increases monotonically with SEI, from ∼0% at ∼13 kJ L^–1^ to ∼5% at ∼65 kJ L^–1^. In contrast, the empty DBD (Figure 1) achieves 12–27% conversion over SEI ∼27–76 kJ L^–1^, indicating that, for a given SEI, the CeO_2_-packed bed yields a much smaller fraction of electrical energy into productive gas-phase electron–molecule collisions compared to the empty DBD reactor. In packed-bed DBD systems, strong local electric field enhancement at particle contact points is typically associated with intensified microdischarges and higher conversion. The absence of such enhancement here suggests that dielectric polarization of CeO_2_ screens the applied electric field, suppressing localized field intensification near particle contact points and thereby limiting the generation of energetic electrons required for efficient CO_2_ activation.^83^

**Figure 12:**
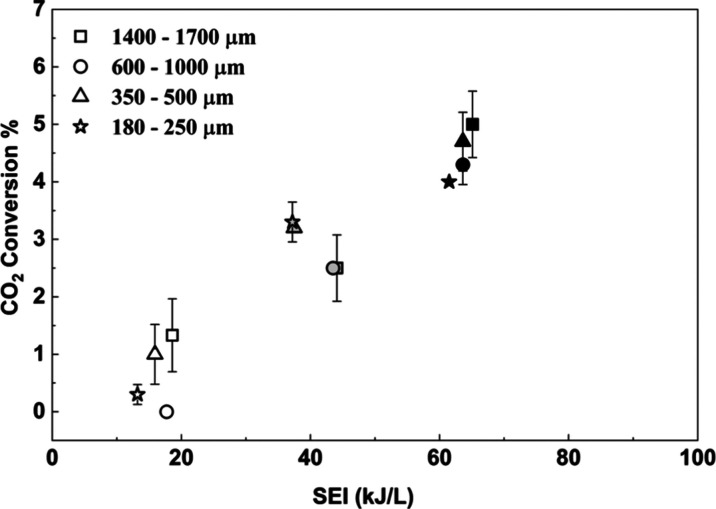
CO_2_ conversion as a function of SEI for CeO_2_-packed bed (CO_2_:H_2_:Ar = 1:3:1, residence time = 6 s, frequency = 23.5 kHz, 1 atm, 298 K) with particle sizes of 180–250 (star), 350–500 (triangle), 600–1000 (circle), and 1400–1700 μm (square). White, gray, and black colors represent input powers of 20, 30, and 40 W, respectively.

Butterworth and Allen have reported that medium dielectric constant materials, such as yttria-stabilized zirconia (YSZ, *ε*_r_ = 25, comparable to CeO_2_), ignite in a polar regime characterized by a homogeneous glow at the dielectric-YSZ interface.^53^ At higher plasma powers, this transitions into a polar-confined streamer regime marked by unstable streamers.^53^ Following this, we employed an optical chamber equipped with a high-speed optical camera to characterize the discharge behavior (details in Section 2.4).

As shown in the supplementary videos for CeO_2_ pellets, a weak, homogeneous glow discharge formed at an applied voltage of 18 kV (Figure S12) and was maintained until applied voltage reached 37 kV. Beyond this threshold, the discharge transitioned into unstable streamers, consistent with the behavior reported by Butterworth and Allen.^53^ Consequently, the CeO_2_-packed bed primarily sustains a weak homogeneous plasma that supports fewer high-energy electron–molecule collisions resulting in low CO_2_ conversion. The particle size influences the extent of plasma formation and propagation by altering the interparticle void distribution. However, because CeO_2_ does not promote surface streamers or localized partial discharges at the operating input powers, the effect of particle size on discharge behavior remains minimal. As a result, CO_2_ conversion across all particle sizes remains nearly identical, despite variations in plasma power and corresponding interparticle void spaces. Thus, at comparable specific energy input (SEI), the dependence on pellet size is also weak, with CO_2_ conversion reaching approximately 5% at a plasma power of ∼21 W (Figure S11). This weak plasma regime correlates with the very low CH_4_ selectivity observed (Figure S13), indicating that the dominant discharge mode under these conditions is homogeneous rather than surface streamers or localized partial discharges, thus providing insufficient energetic electron density to drive extensive hydrogenation reactions.

Unlike in the empty DBD reactor where plasma power is similar for different input powers, packed CeO_2_ shows plasma power variations at different input powers (Figure S11). This is due to changes in the plasma formation and propagation mechanisms with varying interparticle voids for different particle sizes with irregular shapes. Figure 13 and Table 3 summarize the electrical characteristics extracted from the Lissajous plots at an input power of 20 W for empty-DBD (residence time 7.5 s) and CeO_2_-packed beds (residence time 6 s) with different particle sizes. Introducing CeO_2_ pellets substantially alters the electrical behavior of the reactor. Compared with the empty DBD (plasma power = 9.0 W at *V*_pp_ = 13 kV), all packed beds exhibit smaller plot areas and therefore lower plasma power (4.4–6.2 W). The effect is particle size dependent: As particle size decreases, the dissipated plasma power decreases, with the 180–250 μm bed giving the lowest power (4.4 W). Notably, the smallest particle bed requires ∼15 kV yet dissipates roughly half the power of the empty reactor, whereas larger particles operate at similar voltages but yield higher powers (5.3–6.2 W). These trends indicate that, under identical input power, smaller particles increase the capacitive loading (higher *C*_cell_) and reduce interparticle void gaps, shifting more of the applied voltage across the dielectric phase and limiting the gas-phase field, which in turn hampers discharge formation and lowers plasma power. As the input power increases, similar trends in plasma power and *V*_pp_ were observed (see Figure S11 and Tables S6 and S7).

**Figure 13:**
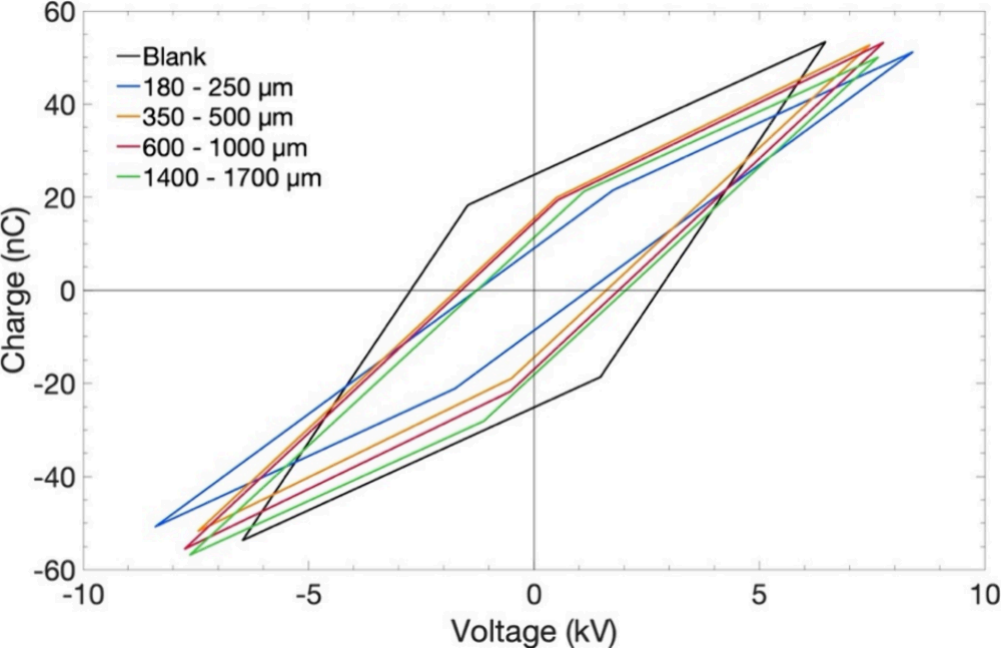
Fitted Lissajous parallelograms for empty-DBD (blank) and CeO_2_-packed bed reactor (CO_2_:H_2_:Ar = 1:3:1, input power = 20 W, feed = 20 cm^3^ min^–1^, frequency = 23.5 kHz, 1 atm, 298 K) with pellet sizes of 180–250, 350–500, 600–1000, and 1400–1700 μm.

**Table 3:** Calculated Discharge Characteristics Obtained from the Instantaneous Current Profiles and Fitted Lissajous Figures for the Empty DBD Reactor and CeO_2_-Packed Bed Reactor with Varying Particle Sizes at Input Power of 20 W and Total Flow Rate of 20 cm^3^ min^–1^

Particle size (μm)	Plasma power (W)	*V*_pp_ (kV)	ζ_diel_ (pF)	*C*_cell_ (pF)	*α*	*V*_bur_ (kV)	*Q*_dis_ (nC)	*N*_md_
	9.0 ± 0.1	12.6 ± 0.1	12.7 ± 0.1	4.4 ± 0.4	0.5	3.1	71 ± 2	21 ± 2
180–250	4.4 ± 0.2	14.9 ± 0.5	8.1 ± 0.9	4.6 ± 0.7	0.8	2.3	69 ± 2	6 ± 0
350–500	5.3 ± 0.1	14.7 ± 0.1	9.3 ± 0.6	4.8 ± 0.1	0.7	2.4	78 ± 3	16 ± 1
600–1000	5.9 ± 0.4	14.5 ± 0.4	9.6 ± 0.4	4.7 ± 0.4	0.7	2.3	84 ± 0	20 ± 1
1400–1700	6.2 ± 0.2	14.5 ± 0.4	10.4 ± 0.3	4.2 ± 0.7	0.7	2.3	84 ± 2	16 ± 1

In agreement with this interpretation, the CeO_2_-packed beds exhibit a lower ζ_diel_ (8.1–10.4 pF) than the empty DBD reactor (12.7 pF), while *C*_cell_ remains nearly constant at ∼4–5 pF across all conditions. For particle sizes ≥300 μm, ζ_diel_ ≈ 9 pF with *α* = 0.7, whereas the 180–250 μm bed shows a slightly lower ζ_diel_ = 8 pF and higher *α* = 0.8, indicating greater spatial confinement of the discharge and less charge transfer between the electrodes. At higher plasma powers, *α* values decrease, indicating more complete discharge with increased charge transfer across the gap. Moreover, both *Q*_dis_ and *N*_md_ are also slightly lower at smaller particle size and lower input powers, whereas it remains nearly constant at 40 W. Because the discharge behavior remains similar across the different particle sizes, the particle size exerts only a weak influence on CO_2_ conversion, consistent with the nearly constant α, *Q*_dis_, and *N*_md_ observed for all packed beds.

In CeO_2_-packed beds, *V*_bur_ is similar for all particle sizes at all input powers. The burning voltage is influenced by the packing material, particle size, and applied voltage at constant gas composition. When the applied electric field is insufficient, reducing particle size enhances partial discharging because discharges cannot easily ignite within the increasingly confined voids of the bed. This effect manifests as an increase in reactor burning voltage with decreasing particle size. However, it has also been noted that *V*_bur_ becomes less reliable as an indicator when extensive partial discharging occurs.^53^

## 4 Conclusions

In this work, we systematically explored the impact of dielectric packing properties on discharge behavior, CO_2_ conversion, and product selectivity in nonthermal plasma-assisted CO_2_ hydrogenation. We demonstrate that Al_2_O_3_ enables highest CO_2_ conversion (up to 42%) due to its low dielectric constant, primarily driven by efficient gas-phase dissociation chemistry. In contrast, high-*ε*_r_ materials BaTiO_3_, TiO_2_, and CeO_2_ yielded lower CO_2_ conversions (<10%), even lower than those for the empty-DBD. Among these, BaTiO_3_ showed higher selectivity toward CH_4_ and C_2+_ products despite lower CO_2_ conversion. CeO_2_ conversely demonstrated lowest CO_2_ conversions, likely due to the absence of surface or contact-anchored streamers, instead sustaining a homogeneous glow discharge at applied voltages below 37 kV, as assessed using a custom-built in situ plasma characterization chamber. This discharge morphology was further supported by the negligible impact of particle size on performance and the consistently low CO_2_ conversion observed.

Our results also highlight the necessity of operating under full plasma discharge conditions when evaluating intrinsic catalytic activity. Partial discharges can lead to underestimation or misrepresentation of the discharge characteristics and reactor performance. Estimations of discharge behavior were guided by the Peeters model, a widely adopted framework, though it remains a simplification of the complex and dynamic plasma environment. Real-time quantification of active plasma zones remains inherently challenging due to spatial and temporal nonuniformities, making precise plasma–catalyst interaction analysis a continuing experimental hurdle.

Overall, our findings demonstrate that the dielectric constant not only dictates electric field localization and discharge morphology but also governs the dominance of gas-phase dissociation versus hydrogenation pathways. These insights provide a foundation for the rational design of plasma-catalyst systems optimized for selective CO_2_ hydrogenation.

## Supplementary Material









## Notes

Plasma power, steady-state voltage, Lissajous figures, CH_4_ selectivities, and calculated discharge characteristics for empty DBD; packed bed porosity, residence times, calculated discharge characteristics, plasma power, CH_4_ selectivity, and instantaneous current profiles for packed beds of different oxides and packed beds of CeO_2_ with varied particle sizes PDF
  High-speed video of plasma discharge over CeO_2_ pellets at 18 kV (a weak and relatively homogeneous glow discharge) (MP4)
High-speed video of plasma discharge over CeO_2_ pellets at 37 kV (unstable filamentary streamers) (MP4)
High-speed video of plasma discharge over CeO_2_ pellets as voltage is increased from 18 to 37 kV (MP4)
