# Supplementary material for: CO2 Hydrogenation Using Nonthermal Plasma: Effects of Packed Beds on Plasma Discharge Behavior
Source: Ind Eng Chem Res. 2026 Feb 23;65(9):4761–74. doi: 10.1021/acs.iecr.5c04396 (PMC12983297; doi:10.1021/acs.iecr.5c04396)
Supplement: Supplementary file 1 [file ie5c04396_si_001.pdf]

Supporting Information

for

CO_2 hydrogenation using non-thermal plasma: Effects of packed beds on plasma discharge behavior

Sathya M. Perera[a], Berkay Ekinci[b], Sven G. Bilén[b,c], Sean D. Knecht [c]*, Gina Noh[d]*

[a] Department of Chemistry, The Pennsylvania State University, University Park, Pennsylvania, 16802, United States

[b] *School of Electrical Engineering and Computer Science*, The Pennsylvania State University, University Park, Pennsylvania, 16802, United States

[c] *School of Engineering Design and Innovation*, The Pennsylvania State University, University Park, Pennsylvania, 16802, United States

[d] Department of Chemical Engineering, The Pennsylvania State University, University Park, Pennsylvania, 16802, United States

*Email: gnoh@psu.edu, sdk149@psu.edu

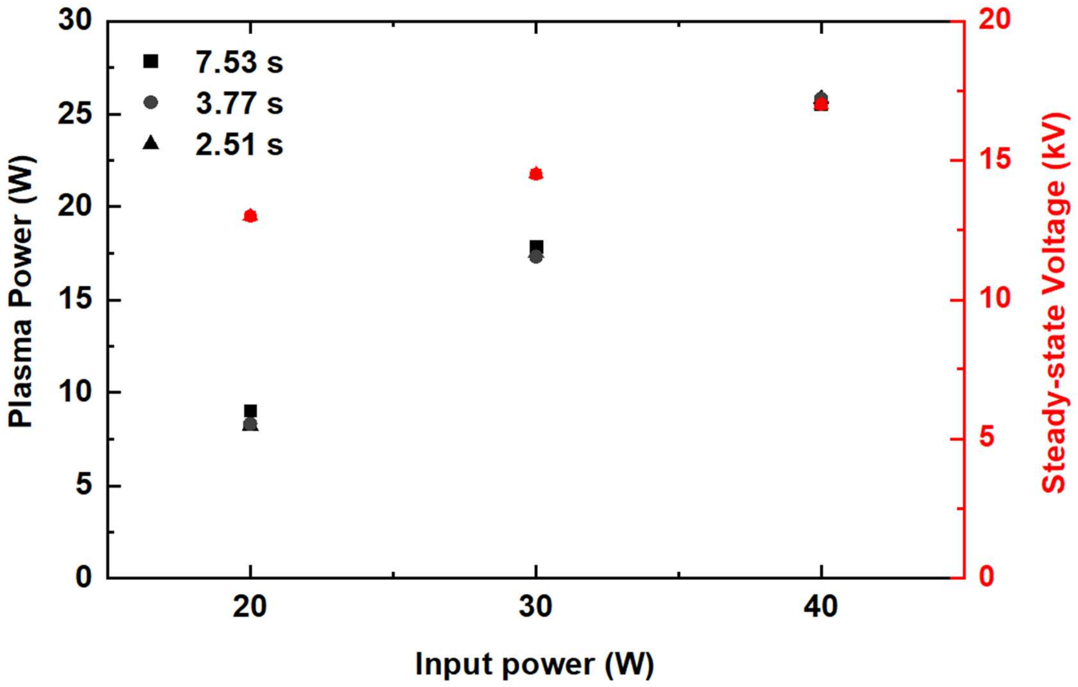

Figure S1. Plasma power (primary ordinate) and steady-state voltage (secondary ordinate) as a function of input power for an empty DBD reactor (CO_2:H_2:Ar = 1:3:1, frequency = 23.5 kHz). Data points represent different residence times, as indicated in the legend; three data points are present for each input power for plasma power and for steady-state voltage, respectively.

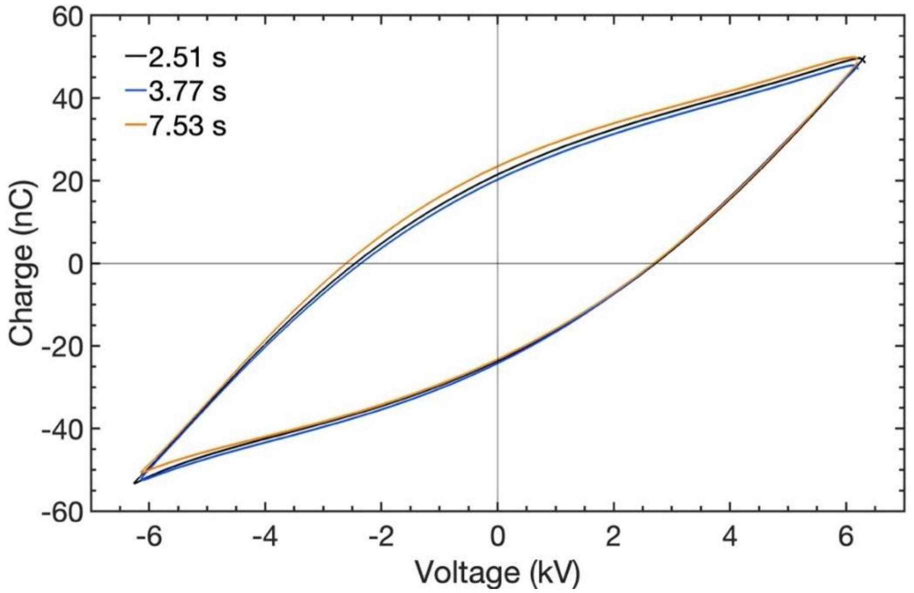

Figure S2. Lissajous plots at different residence times of 2.51 s, 3.77 s, and 7.53 s for an empty-DBD reactor (CO_2:H_2 Ar = 1:3:1, frequency = 23.5 kHz, 1 atm, 298 K) at an input power of 20 W. The calculated area of the plot corresponds to plasma power of 9 W.

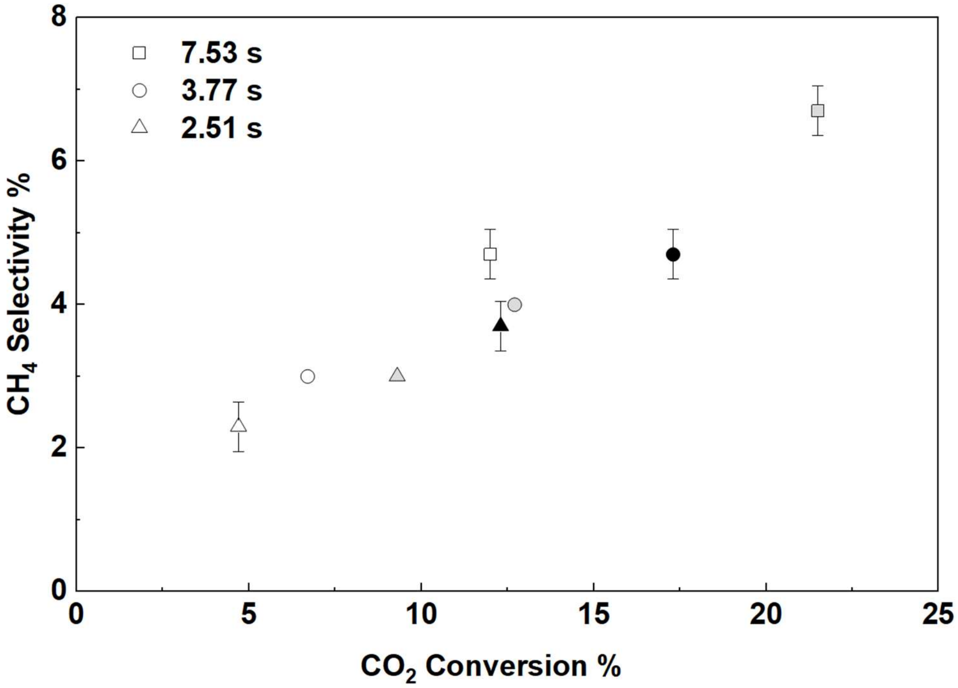

Figure S3. CH_4 selectivity as a function of CO_2 conversion for an empty DBD reactor (CO_2:H_2 Ar = 1:3:1, frequency = 23.5 kHz, 1 atm, 298 K). Squares, circles, and triangles represent residence times of 7.53, 3.77, and 2.51 s and white, grey and black represent 8, 18, and 26 W, respectively.

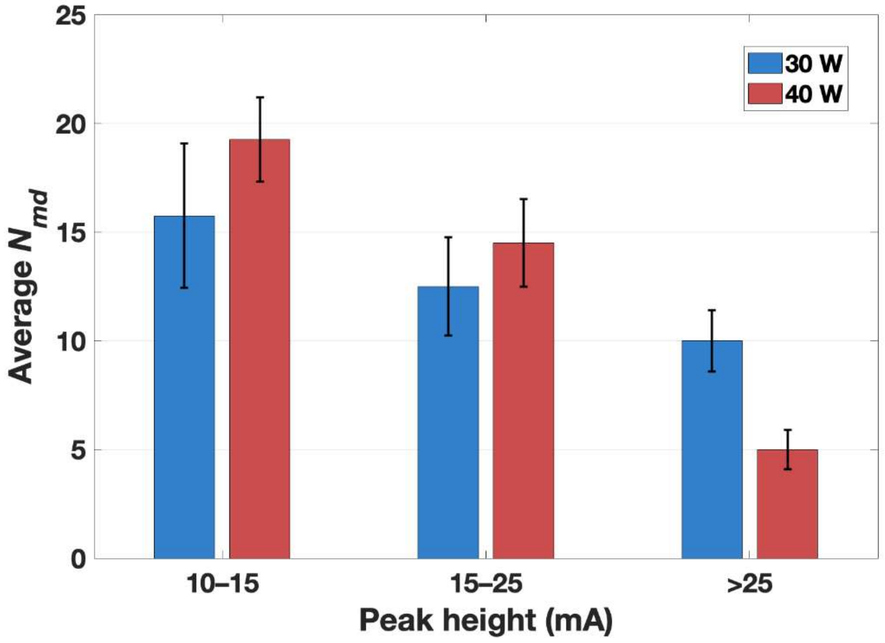

Figure S4. Statistical comparison of micro discharge peak-height distributions at input powers of 30 W and 40 W for an empty DBD reactor.

Residence time and Packing porosity

The residence time for the empty DBD reactor is calculated according to Equation 1

$$\text{Residence time, } \tau \text{ (s)} = \frac{V_{annular}}{v_0} \qquad \text{(Equation S1)}$$

where, $V_{annular}$ is the volume of the annulus for the 5 cm long plasma zone (2.51 cm^3), and v_0 is the total flow rate. For the packed reactor, τ was corrected with packing porosity ($\varepsilon_{packing}$), as seen in Equation 2:

$$\text{Residence time, } \tau \text{ (s)} = \frac{V_{annular}}{v_0} \cdot \varepsilon_{packing} \qquad \text{(Equation S2)}$$

where, $\varepsilon_{packing}$ is the void volume relative to the volume of the annulus ($V_{void}/V_{annular}$). V_{void} was calculated as follows:

$$V_{void} = V_{annular} - V_{solid} \qquad \text{(Equation S3)}$$

where, $V_{annular}$ = 2.5 cm^3 and V_{solid} was calculated by mass of the packed material:

$$V_{solid} = \frac{mass\ (g)}{Bulk\ density\ (g\ cm^{-3})} \qquad \text{(Equation S4)}$$

The $\varepsilon_{packing}$ and residence time for different packing materials are provided in the Table S2. The mass required to pack 5 cm of the fixed annular region of the DBD is varied with the dielectric material: 1.5 g for Al_2O_3 (4 g cm^{-3}); 4 g for CeO_2 (7 g cm^{-3}), 2.8 g for TiO_2 (4.3 g cm^{-3}); and 6.25 g for $BaTiO_3$ (6.1 g cm^{-3}).

Table S1. Calculated discharge characteristics obtained from the instantaneous current profiles and fitted Lissajous figures for the empty DBD reactor at residence times of 7.53 s, 3.77 s, and 2.51 s at an input power of 20 W, as determined by a MATLAB script.

Residence time (s)	Plasma power (W)	V_{pp} (kV)	ζ_{diel} (pF)	C_{cell} (pF)	α	V_{bur} (kV)	Q_{dis} (nC)	N_{md}
2.51	8.6 ± 0.1	12.5 ± 0.2	13.0 ± 0.2	4.2 ± 0.1	0.5	3.1	75 ± 1	20 ± 1
3.77	8.6 ± 0.2	12.4 ± 0.1	12.9 ± 0.5	4.2 ± 0.2	0.5	3.1	75 ± 1	23 ± 1
7.53	9.0 ± 0.1	12.6 ± 0.1	12.7 ± 0.1	4.4 ± 0.4	0.5	3.1	71 ± 2	21 ± 2

Table S2. BET surface area, BJH pore characteristics, reactor porosity and residence times of different dielectric materials of packed beds for particle size of 600-1000 μm at a total flow rate of 20 mL min^{-1} and input power of 20 W.

Dielectric material	Surface area (m^2 g^{-1})	Pore volume (cm^3 g^{-1})	Average pore size (nm)	Reactor porosity	Residence time (s)
Al_2O_3	146	1.06	24.7	0.85	6.40
CeO_2	87.2	0.23	10.7	0.77	5.81
TiO_2	104	0.55	23.0	0.74	5.56
$BaTiO_3$	6.1	0.025	19.1	0.59	4.45

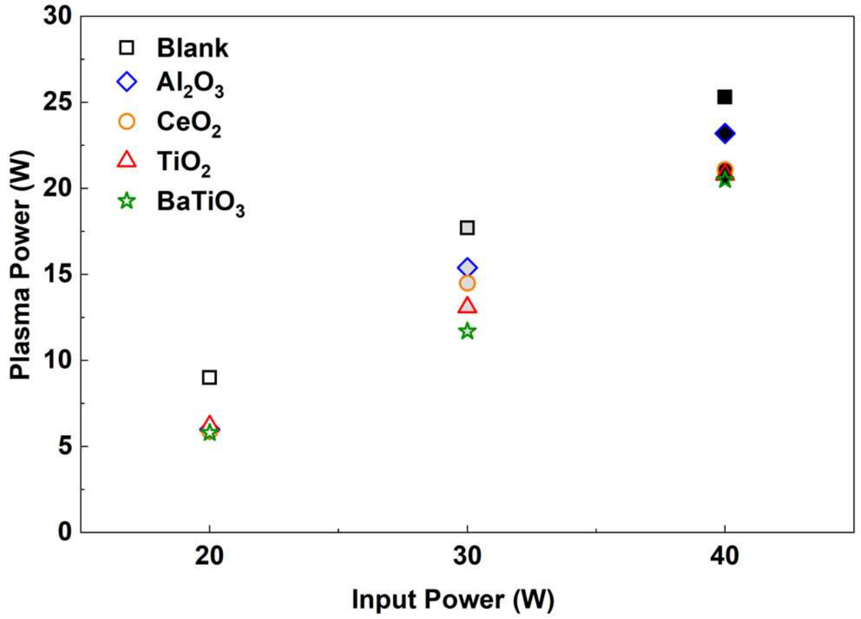

Figure S5. Plasma power and as a function of input power for packed bed reactor (CO_2:H_2:Ar = 1:3:1, feed = 20 mL min^{-1}, frequency = 23.5 kHz, particle size = 600-1000 µm, 1 atm, 298 K). Squares, diamonds, circles, triangles, and stars represent empty-DBD, Al_2O_3, CeO_2, TiO_2, and $BaTiO_3$, and while white, grey, and black represent input powers of 20, 30 and 40 W, respectively.

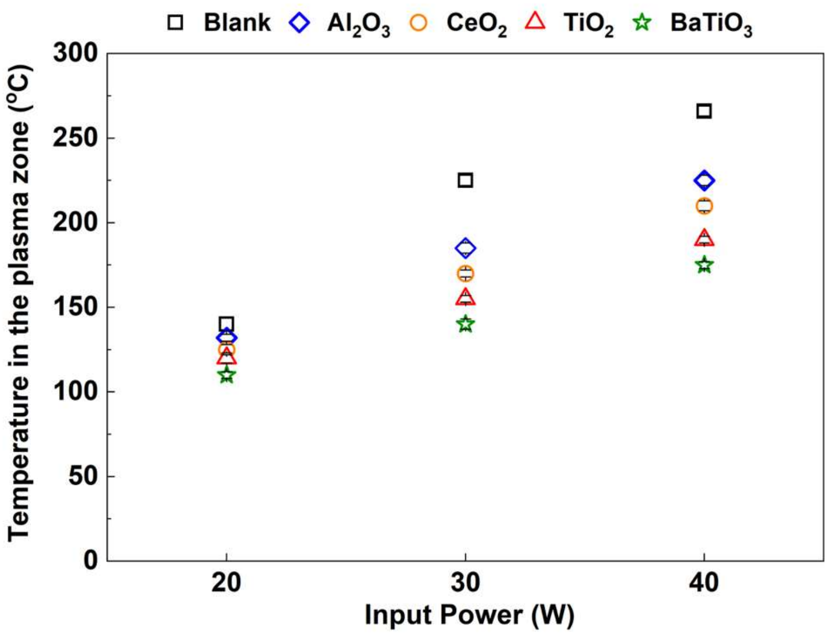

Figure S6. Measured temperature inside the plasma zone at different input powers for the empty DBD reactor and packed-bed configurations of Al_2O_3, CeO_2, TiO_2, and $BaTiO_3$.

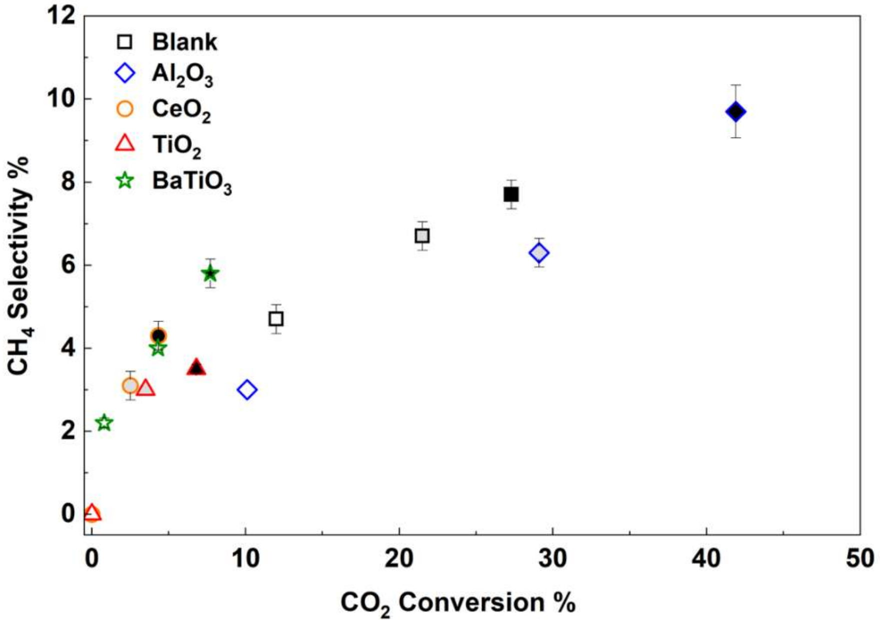

Figure S7. CH_4 selectivity as a function of SEI for packed bed reactor (CO_2:H_2:Ar = 1:3:1, feed = 20 mL min^{-1}, frequency = 23.5 kHz, particle size = 600-1000 µm, 1 atm, 298 K). Squares, diamonds, circles, triangles, and stars represent empty-DBD, Al_2O_3, CeO_2, TiO_2, and $BaTiO_3$, and while white, grey, and black represent input powers of 20, 30 and 40 W, respectively.

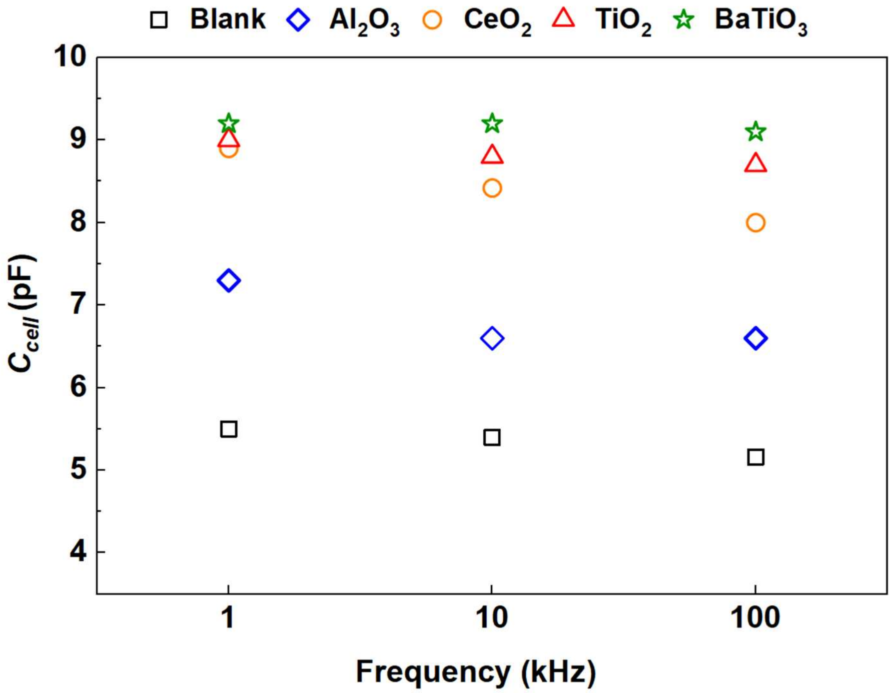

Figure S8. C_{cell} measured by LCR meter under plasma-off conditions as a function of frequency for the empty DBD reactor and packed-bed configurations of Al_2O_3, CeO_2, TiO_2, and $BaTiO_3$.

Table S3. Calculated discharge characteristics obtained from the instantaneous current profiles and fitted Lissajous figures for the empty DBD reactor and reactor packed with Al_2O_3, CeO_2, TiO_2, and $BaTiO_3$ at input power of 30 W, as determined by a MATLAB script.

Dielectric Material	Plasma power (W)	V_{pp} (kV)	ζ_{diel} (pF)	C_{cell} (pF)	α	V_{bur} (kV)	Q_{dis} (nC)	N_{md}
-	17.7 ± 0.3	14.2 ± 0.2	19.1 ± 0.4	3.9 ± 0.3	0.1	3.1	128 ± 3	40 ± 1
Al_2O_3	15.4 ± 0.4	15.4 ± 0.1	14.3 ± 0.5	4.1 ± 0.4	0.4	3.4	98 ± 3	34 ± 1
CeO_2	14.5 ± 0.4	16.8 ± 0.5	12.6 ± 0.4	4.1 ± 0.4	0.5	3	135 ± 2	34 ± 0
TiO_2	13.1 ± 0.2	16.7 ± 0.1	16.3 ± 0.4	5.2 ± 0.3	0.3	1.5	214 ± 2	32 ± 1
$BaTiO_3$	11.7 ± 0.2	17.9 ± 0.0	13.4 ± 0.0	6.4 ± 0.2	0.5	2.1	166 ± 1	37 ± 2

Table S4. Calculated discharge characteristics obtained from the instantaneous current profiles and fitted Lissajous figures for the empty DBD reactor and reactor packed with Al_2O_3, CeO_2, TiO_2, and $BaTiO_3$ at input power of 40 W, as determined by a MATLAB script.

Dielectric Material	Plasma power (W)	V_{pp} (kV)	ζ_{diel} (pF)	C_{cell} (pF)	α	V_{bur} (kV)	Q_{dis} (nC)	N_{md}
-	25.3 ± 0.3	16.4 ± 0.2	20.8 ± 0.2	4.3 ± 0.2	0	3	175 ± 1	38 ± 1
Al_2O_3	23.2 ± 0.4	17.2 ± 0.1	17.9 ± 0.4	4.1 ± 0.1	0.2	3.6	147 ± 2	43 ± 1
CeO_2	21.1 ± 0.2	19.4 ± 0.0	17.8 ± 0.3	6.7 ± 0.5	0.2	2.3	240 ± 2	22 ± 1
TiO_2	20.8 ± 0.2	19.7 ± 0.1	20.1 ± 0.1	8.8 ± 0.2	0.1	1.8	289 ± 2	26 ± 2
$BaTiO_3$	20.5 ± 0.4	19.3 ± 0.2	18.8 ± 0.2	7.2 ± 0.4	0.2	2.2	255 ± 2	23 ± 2

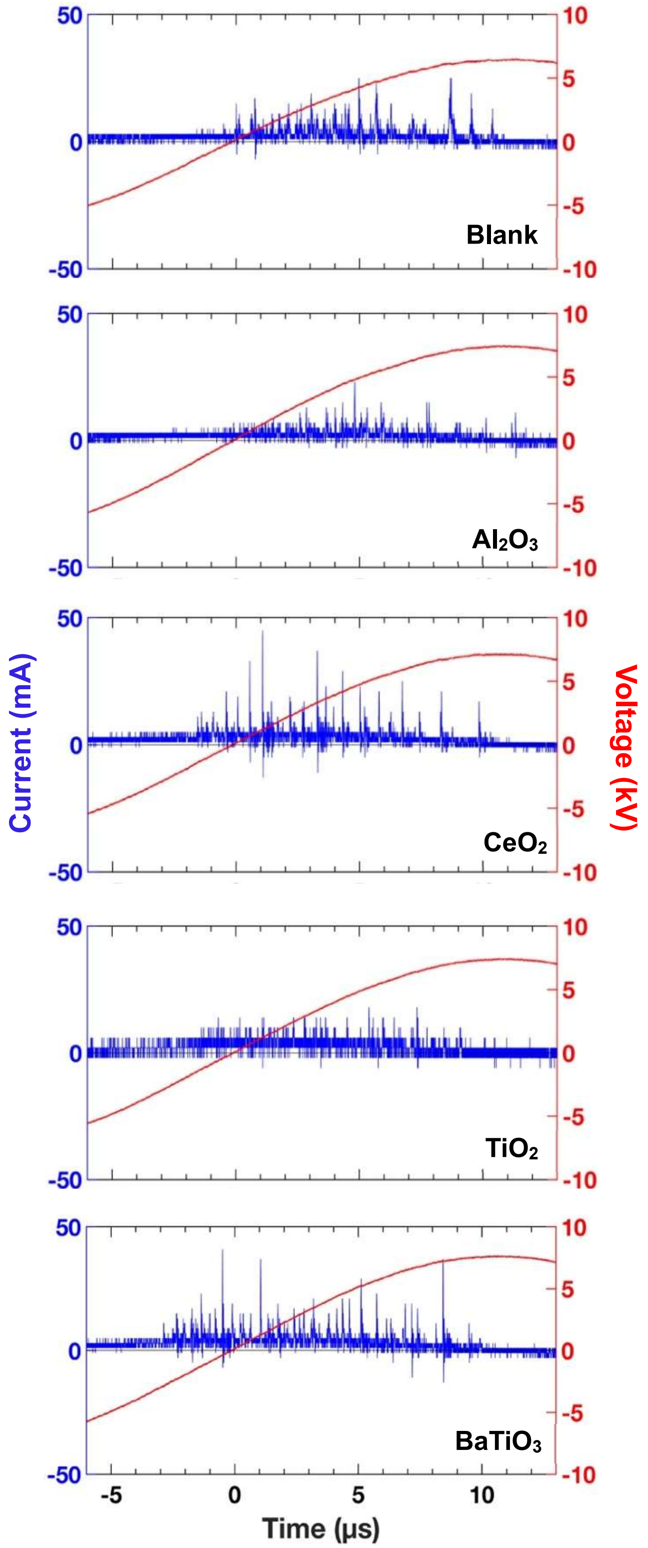

Figure S9. Instantaneous current profiles during the positive half-cycle of the applied voltage for the empty DBD reactor and packed-bed configurations of Al_2O_3, CeO_2, TiO_2, and $BaTiO_3$ at an input power of 20 W.

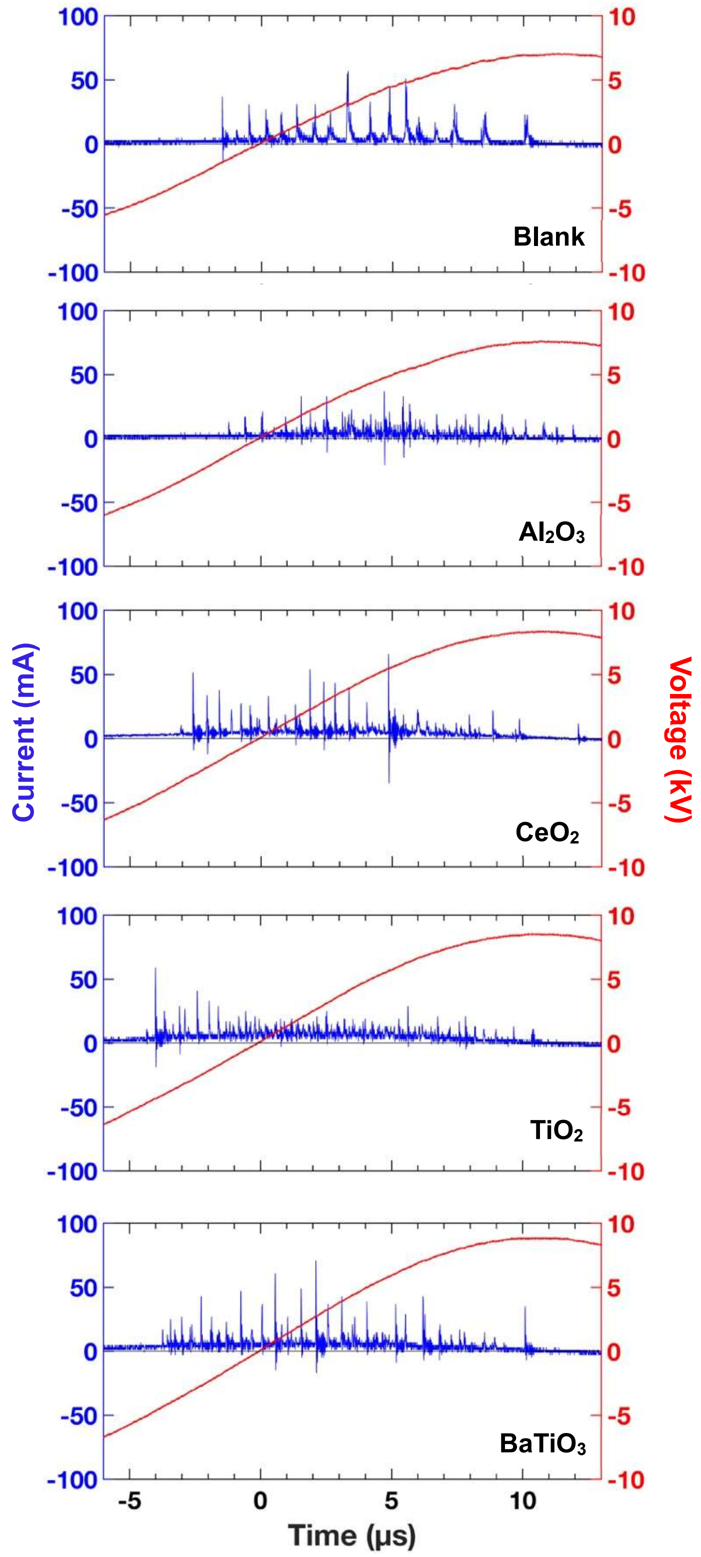

Figure S10. Instantaneous current profiles during the positive half-cycle of the applied voltage for the empty DBD reactor and packed-bed configurations of Al_2O_3, CeO_2, TiO_2, and $BaTiO_3$ at an input power of 30 W.

Table S5. Reactor porosity and residence times of different particle sizes of CeO_2-packed beds at a total flow rate of 20 mL min^{-1} and input power of 20 W.

Pellet size (μm)	Loading mass (g)	Packing porosity	Residence time (s)
1400 -1700	4.00	0.79	5.81
600 -1000	4.00	0.79	5.81
350 - 500	3.75	0.80	5.92
180 – 250	3.50	0.82	6.03

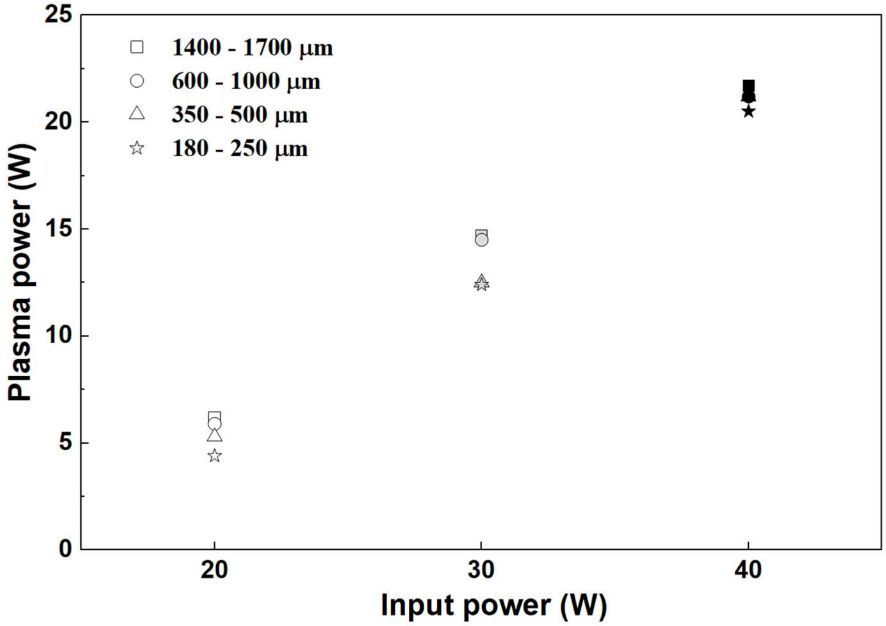

Figure S11. Plasma power and as a function of input power for CeO_2-packed beds (CO_2:H_2:Ar = 1:3:1, residence time = 6 s, frequency = 23.5 kHz). Squares, circles, triangles, and stars represent particle sizes of 180-250, 350-500, 600-1000, and 1400-1700 μm, and while white, grey, and black represent input powers of 20, 30 and 40 W, respectively.

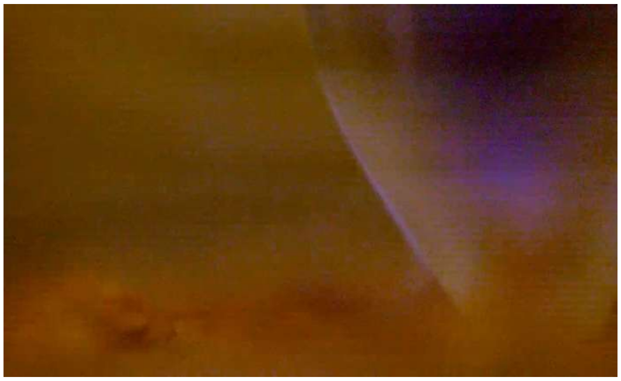

Figure S12. High-speed image of plasma discharge over CeO_2 pellets captured using a Phantom TMX 7510 (serial no. 311136). The recording was conducted at a resolution of 1280 × 760 pixels and a frame rate of 300 frames per second, with an exposure time of 3300 μs and an exposure index of 25,000.

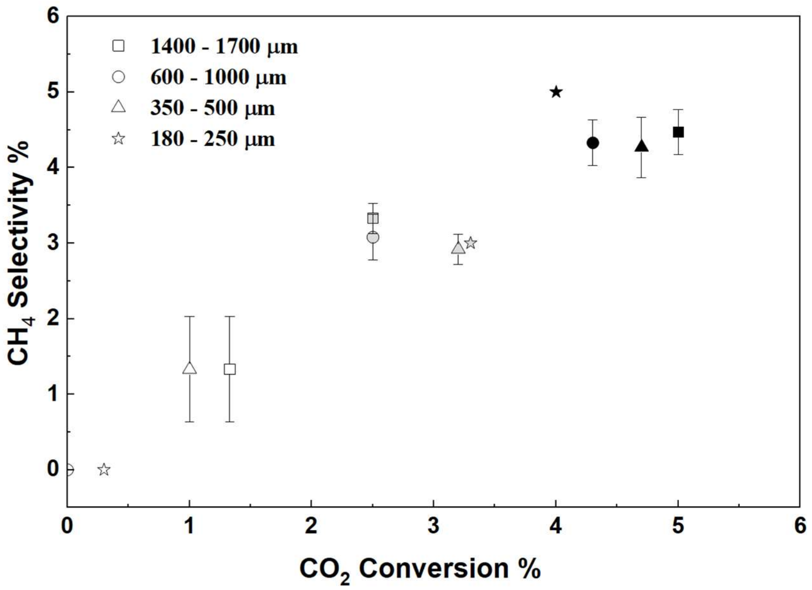

Figure S13. CH_4 selectivity as a function of CO_2 conversion CeO_2-packed beds (CO_2:H_2:Ar = 1:3:1, residence time = 6 s, frequency = 23.5 kHz). Squares, circles, triangles, and stars represent particle sizes of 180-250, 350-500, 600-1000, and 1400-1700 μm, and while white, grey, and black represent input powers of 20, 30 and 40 W, respectively.

Table S6. Calculated discharge characteristics obtained from the instantaneous current profiles and fitted Lissajous figures for the empty DBD reactor and CeO_2-packed bed reactor with varying particle size at input power of 30 W, as determined by a MATLAB script.

Particle size (μm)	Plasma power (W)	V_{pp} (kV)	ζ_{diel} (pF)	C_{cell} (pF)	α	V_{bur} (kV)	Q_{dis} (nC)	N_{md}
-	17.7 ± 0.3	14.2 ± 0.2	19.1 ± 0.4	3.9 ± 0.3	0.1	3.1	128 ± 3	40 ± 1
180 - 250	12.4 ± 0.1	16.2 ± 0.7	12.2 ± 0.6	4.3 ± 0.1	0.5	2.3	122 ± 1	27 ± 1
350 - 500	12.5 ± 0.6	16.6 ± 0.1	12.2 ± 0.9	4.2 ± 0.5	0.6	2.2	130 ± 3	35 ± 1
600 - 1000	14.5 ± 0.4	16.8 ± 0.5	12.6 ± 0.4	4.1 ± 0.4	0.5	2.4	135 ± 2	34 ± 1
1400 - 1700	14.7 ± 0.6	16.8 ± 0.6	13.6 ± 0.1	4.2 ± 0.0	0.5	2.4	141 ± 3	29 ± 2

Table S7. Calculated discharge characteristics obtained from the instantaneous current profiles and fitted Lissajous figures for the empty DBD reactor and CeO_2-packed bed reactor with varying particle size at input power of 40 W, as determined by a MATLAB script.

Particle size (μm)	Plasma power (W)	V_{pp} (kV)	ζ_{diel} (pF)	C_{cell} (pF)	α	V_{bur} (kV)	Q_{dis} (nC)	N_{md}
-	25.3 ± 0.3	16.4 ± 0.2	20.8 ± 0.2	4.3 ± 0.2	0	3	175 ± 1	38 ± 1
180 - 250	20.5 ± 0.6	19.8 ± 0.8	18.7 ± 0.9	6.1 ± 0.1	0.2	2.1	245 ± 3	19 ± 1
350 - 500	21.2 ± 0.8	19.3 ± 0.6	17.9 ± 0.0	6.3 ± 0.9	0.2	2.3	243 ± 4	23 ± 1
600 - 1000	21.2 ± 0.2	19.4 ± 0.0	17.8 ± 0.3	6.7 ± 0.5	0.2	2.3	240 ± 2	22 ± 1
1400 - 1700	21.7 ± 0.5	19.8 ± 0.1	18.5 ± 0.5	7.3 ± 0.1	0.2	2.4	241 ± 3	23 ± 1
